# CD4/CD8/Dendritic cell complexes in the spleen: CD8^+^ T cells can directly bind CD4^+^ T cells and modulate their response

**DOI:** 10.1371/journal.pone.0180644

**Published:** 2017-07-07

**Authors:** Aleksandr Barinov, Alessia Galgano, Gerald Krenn, Corinne Tanchot, Florence Vasseur, Benedita Rocha

**Affiliations:** 1 INSERM, U1020, Faculté de Médecine René Descartes, Paris, France; 2 INSERM, U970, Université Paris Descartes, Centre de recherche Cardiovasculaire à l’HEGP, Paris, France; Maisonneuve-Rosemont Hospital, CANADA

## Abstract

CD4^+^ T cell help to CD8^+^ T cell responses requires that CD4^+^ and CD8^+^ T cells interact with the same antigen presenting dendritic cell (Ag^+^DC), but it remains controversial whether helper signals are delivered indirectly through a licensed DC and/or involve direct CD4^+^/CD8^+^ T cell contacts and/or the formation of ternary complexes. We here describe the first *in vivo* imaging of the intact spleen, aiming to evaluate the first interactions between antigen-specific CD4^+^, CD8^+^ T cells and Ag^+^DCs. We show that in contrast to CD4^+^ T cells which form transient contacts with Ag^+^DC, CD8^+^ T cells form immediate stable contacts and activate the Ag^+^DC, acquire fragments of the DC membranes by trogocytosis, leading to their acquisition of some of the DC properties. They express MHC class II, and become able to present the specific Marilyn peptide to naïve Marilyn CD4^+^ T cells, inducing their extensive division. *In vivo*, these CD8^+^ T cells form direct stable contacts with motile naïve CD4^+^ T cells, recruiting them to Ag^+^DC binding and to the formation of ternary complexes, where CD4^+^ and CD8^+^ T cells interact with the DC and with one another. The presence of CD8^+^ T cells during *in vivo* immune responses leads to the early activation and up-regulation of multiple functions by CD4^+^ T lymphocytes. Thus, while CD4^+^ T cell help is important to CD8^+^ T cell responses, CD8^+^ T cells can interact directly with naïve CD4^+^ T cells impacting their recruitment and differentiation.

## Introduction

CD4^+^ T cells have a fundamental role in CD8^+^ T cell responses. They potentiate primary immune responses, enhance the expansion of antigen-specific CD8^+^ T cells, and are fundamental for the generation of CD8^+^ memory cells [1]. In the absence of CD4^+^ help, primed CD8^+^ T cells never develop the enhanced capacity to divide and secrete cytokines characteristic of CD8^+^ memory cells [1–3]. Repeated boosting cannot overcome this CD4^+^ requirement, successive CD8^+^ responses maintaining all the characteristics of the primary immune response [2].

Although CD4^+^ and CD8^+^ T cells recognize different peptides, it is well established that in order to provide help, antigen-specific CD4^+^ and CD8^+^ T cells must interact with the same antigen presenting cell (APC) presenting both the CD4 and CD8 peptides derived from the same antigen (Ag) [1, 4]. How the interactions between these three cell types occur is yet a subject of debate. It was argued that naïve antigen-specific CD4^+^ and CD8^+^ T cells, which are infrequent in the naïve pool, would have a very low probability to bind simultaneously to the same antigen presenting dendritic cell (Ag^**+**^DC). Thus, a first model was proposed where CD4^+^ T cells would first encounter and induce the licensing of the Ag^**+**^DC, which subsequently would become able to potentiate CD8^+^ T cell responses [4].

Recent studies indicate that simultaneous interactions between antigen specific CD4^+^ and CD8^+^ T cells and the same Ag^**+**^DC could be more frequent than envisaged previously. In the lymph nodes (LN), DCs accumulate at strategic locations that facilitate T/Ag^+^DC interactions: around high endothelium venules (HEV) [5] or in the T cell zone, where they were reported to migrate after innate stimulation [6, 7]. *In vivo* imaging showed that T lymphocytes are highly motile, and that activated DCs protrude dendrites allowing the screening of 500–5000 lymphocytes/hour [6, 8]. Moreover, upon immunization of CD4^+^ T cells with their specific peptides and adjuvants, the amount of CCL3 and CCL4 increases locally, and CD8^+^ T cells accumulate in the place where CD4^+^ T cells are located [9]. We have shown that secretion of these chemokines is not an exclusive property of activated CD4^+^ T cells. After infection with *Listeria monocytogenes* (LM) expressing ovalbumin (OVA), OT-I OVA-specific CD8^+^ T cells secrete higher amounts of CCL3 and CCL4 than OT-II OVA-specific CD4^+^ T cells immunized simultaneously [10]. These chemokines are produced immediately after stimulation. 56 of these CD8^+^ effector T cells injected in to a lymph node are able to recruit more than 10^7^ cells in 24 hours. CD8^+^ T cell priming also induces a transient ten-fold increase in the local S1P concentration, which leads to the local retention of all recruited cells for 24–48 hours [10], as it was described in the “shut-down-phase” of lymphocyte trapping [11]. These multiple events may give ample opportunity to motile antigen-specific CD4^+^ and CD8^+^ T cells to bind rare Ag^**+**^DCs.

It is currently assumed that both CD4^+^ and CD8^+^ T cells only interact directly with Ag^**+**^DC and CD4 help to CD8 responses is indirect, mediated by DC activation [12]. Two recent reports that studied the behavior of T cells and DCs in the intact LN after infection with non-replicating viruses showed that the Ag was first transported by migratory DCs to the LNs, but the initial clustering of CD8^+^ and CD4^+^ T cells occurred on different DC subsets. Although the DC subsets involved in these initial clustering differed in these two reports, both reports demonstrated that after such spatially separate activation of T cells, CD4^+^ and CD8^+^ T cells migrate to and cluster around XCR1^+^DCs that cross present the Ag [13, 14]. In contrast to these results three independent reports using different experimental systems showed that CD4^+^ and CD8^+^ T cells interact directly with one another [1, 15, 16]. By using the male antigen model we showed that CD4 help to CD8^+^ responses does not require CD40 expression by the APCs but depends instead on the expression of CD40 by CD8^+^ T cells, suggesting that CD4^+^/CD8^+^ T cells interact directly [1]. In another model using OVA pulsed DCs, Ahmed et al., showed that CD4^+^ and CD8^+^ T cells interact directly and that these direct CD4^+^/CD8^+^ interactions were necessary and sufficient to provide CD4 help [15]. Lastly, by employing the LCMV infection model Romagnoli et al., demonstrated that the *in vitro* incubation of CD4^+^ T cells recovered from LCMV infected mice with CD8^+^ T lymphocytes was sufficient to provide help and generate CD8^+^ T cell memory [16]. Based on these results Romagnoli et al., proposed that during LCMV infection CD8^+^ T cells would acquire fragments of DC membranes by trogocytosis [17, 18], allowing them to express MHC class II and present the Ag directly to CD4^+^ T cells, but this hypothesis was not directly demonstrated.

Overall, these data suggest that T cell and DC behavior may depend on the nature of the Ag, on the type of DCs presenting the Ag, and on the methods used for Ag administration. Moreover, studies on T cell behavior in the LN [5–9, 12–15, 19–21] and the spleen [22, 23] indicate that T cell motility is much slower in the spleen, i.e., depend on the organ being investigated.

In this study we aimed to determine the first naïve T cells interactions with non-manipulated Ag^+^DCs in the spleen. Using Two-Photon Laser Scanning Microscopy (TPLSM) and confocal microscopy, we show that from 4–7 hours after T cell transfer antigen-specific CD8^+^ T cells form immediate stable contacts with the Ag^+^DC in the red pulp (RP) of the spleen. Upon Ag^**+**^DC binding, the CD8^+^ T cells acquire DC membrane fragments and MHC class II molecules by trogocytosis. *In vitro* MHC class II^+^ CD8^+^ T cells become able to present the specific peptide to naïve CD4^+^ T cells and induce their extensive division. *In vivo* CD8^+^ T cells establish stable complexes with motile naïve CD4^+^ T lymphocytes; recruit them to bind Ag^+^DC and form ternary complexes. The majority of the T/DC complexes found in the spleen are ternary, where both CD4^+^ and CD8^+^ T cells interact with the Ag^+^DC and between themselves. Lastly, the presence of CD8^+^ T cells potentiate naïve CD4^+^ T cell immune responses *in vivo*, accelerating CD4^+^ T cell activation and division, and increasing their expression of multiple mRNAs coding for effector functions, adhesion, and activation molecules. Thus, although CD4^+^ T cells have a major impact on CD8^+^ T cell responses, CD8^+^ T lymphocytes also modulate CD4^+^ T cell immune reactions.

## Materials and methods

### Mice

All mice were 6–8 weeks old Rag2^**-/-**^ C57BL/6 (B6 mice), bred in isolators at the Center for the Development of Advanced Experimental Techniques (Orleans, France). The health status of the colonies was monitored every three months to confirm their specific-pathogen-free conditions. Rag2^**-/-**^ C57BL/6 mice were: female or male Rag2^**-/-**^ mice expressing GFP under chicken beta actin promoter [24]; female mice expressing TCR-αβ transgenes (Tg) specific for the male antigen. Since these mice were on Rag2^-/-^ background they could not rearrange endogenous TCRs. All their T cells only expressed the already rearranged TCR-Tg. These cells were all naïve since they were obtained from female mice that do not express the male Ag. They were either restricted to the MHC class II IA^b^: Monoclonal (Mo) CD4^+^ Marilyn cells [25]; or restricted to the MHC class I H-2D^b^: MoTCR Tg CD8^+^ cells [26]. Their phenotype is shown in S1 Fig. To allow the expression of different fluorochromes by each TCR-Tg type, CD4^+^ T cells were labeled with 10μM CellTrace Violet vital dye (Invitrogen, Carlsbad, CA, USA) before injection. Mice expressing DSRed under the human CD2 promoter (a kind gift from A. Potocnik) were backcrossed into the Mo TCR-Tg CD8^+^ background. This promoter leads to permanent labeling of all T lymphocytes [27]. Experimental mice were monitored daily, and were kept in isolators until imaging or disposal. Mice were sacrificed by carbon dioxide asphyxia. Experimental procedures were approved by the French University Animals Ethics Committee and conducted according to the institutional guidelines of the European Community.

### Immunization protocols

Sub-lethally irradiated (400Rad) Rag2^**-/-**^ female mice were injected i.v. with 0.5×10^6^ Rag2^**-/-**^ GFP^+^ BM cells from female or male mice. Three and a half days later, these mice were injected i.v. with 1.5×10^6^ CellTrace Violet labeled CD4^+^ Marilyn MoTCR-Tg cells alone, or with an equal number of CellTrace Violet^+^ CD4^+^ and DSRed^+^ CD8^+^ MoTCR-Tg cells.

### Antibodies and immunofluorescence analysis

The following MoAb were obtained from BD Pharmingen and used for cell surface staining: APC-labeled anti-CD3 (145-2C11); PerCP-labeled anti-CD4 (RM4-5); BV786-labelled anti-CD4 (GK-1.5); PerCP-labeled anti-CD8 (53–6.7); PE and FITC-labeled anti-TCR Vβ6 (RR4-7); PE-labeled anti-T3.70 (anti-TCR-α Tg); PE or PECy7-labeled anti-CD69 (H1.2F3); APC-labeled anti-CD44 (IM7), APC-Cy7-labelled anti-CD25 (PC61); biotin-labeled anti-CXCR5 (2G8) and PerCP-labeled streptavidin (554064). FITC-labeled anti-T3.70 (anti-TCRα Tg) was obtained from eBioscience (eBioscience, San Diego, CA, USA). Pacific Blue-labeled anti-CD54 (YN1/1.7.4) was obtained from BioLegend. Pacific Blue-labeled anti-CD8β (H35-172) was conjugated in our laboratory using a labeling kit (Invitrogen, Carlsbad, CA, USA), Cells were analyzed in a FACSCantoII or FACSFortesa and sorted in a FACSAria (Becton Dickinson, Franklin Lakes, NJ, USA); data analysis was performed using Flow Jo (TreeStar Inc. Ashland, OR, USA) or the Diva software (Becton Dickinson, Franklin Lakes, NJ, USA).

### Histology and confocal microscopy

Mice were sacrificed by carbon dioxide asphyxia; the spleens were harvested and immersion-fixed in 4% formaldehyde 10% sucrose in PBS for 30 minutes. Fixed organs were washed briefly in PBS and frozen directly in Tissue-Tec OCT (Fisher, Pittsburgh, PA, USA). Frozen tissue blocks were brought to −20°C and 20 μm sections were cut and placed on SuperFrost Ultra Plus adhesion slides (Gerhard Menzel GmbH, Braunschweig, Germany). Slides were air dried for 2 hours, washed in PBS to remove the OCT, mounted in Vectashield Mounting Media (Vector Laboratories, Inc. Burlingame, CA, USA) and cover-slipped. Spleen sections were imaged using a Zeiss LSM700 axioplan confocal microscope equipped with ZEN image acquisition software (Carl Zeiss Micro-Imaging GmbH, Germany); or Leica SP5 microscope equipped with LAS AF software (Leica Microsystems GmbH, Wetzlar, Germany). Low-magnification images were collected using 20x no-immersion plan-apochromat objectives (NA 0.75 for Zeiss LSM700, and NA 0.70 for Leica SP5 microscopes); high-resolution images were collected using 63x oil immersion objectives (NA 1.4). Cell numbers and cell contacts were quantified manually using respectively ImageJ (NIH, Bethesda, Maryland, USA) and IMARIS image processing software (Bitplane AG, Switzerland). For the detection of putative contact zones, we used the Imaris co-localization plug-in, taking in advantage the fact that the bright CellTrace Violet, DsRed and GFP signals resulted in a weak halo of fluorescence, extending the actual cell limits by 0.5–1 μm so that close proximity consistently resulted in co-localized fluorescent signals [28].

### Intravital two-photon laser-scanning microscopy imaging

Mice were anaesthetized with 100mg/kg ketamine/10 mg/kg xylazine i.p. Tail and paw pinching was used to assess the depth of the anesthesia every 30 minutes. After ~40 min, anesthesia was maintained by subcutaneous injections of half doses of anesthetic approximately every 60 min. In all cases, mice were unconscious before starting the procedure and throughout the experiment. To expose the spleen, the abdomen of the animal was shaved, and a ~1.5 cm incision was made in the left flank. We designed a special stage and imaging platform allowing immobilization of the spleen, and preventing that the animal’s respiratory movements would affect imaging. The mouse was placed on this stage and inverted onto a glass coverslip mounted within a custom-made imaging platform and the stage was stabilized on the imaging platform with adhesive tape. Local temperature was maintained at 37°C in a blacked-out environmental chamber of the Lavision Biotec Microscope on an Olympus statif setting (LaVision BioTec GmbH, Germany).

The TPLSM setting was equipped with a Titanium Sapphire laser MaiTai HP from Spectra Physics (Newport Spectra-Physics GmbH, Darmstadt, Germany) tuned to 860 nm. The emitted fluorescence was collected by three PMTs with the following emission filters: 400-480nm, 510-545nm, and 575-630nm. The images in movies were collected using 20x water immersion objective (NA 0.9) typically from 21 z-planes spaced 2.5–6 μm apart between 50 and 150 μm below the capsule. This z-stack image collection was repeated every 60s to create four-dimension data sets for up to 2 h of imaging time. Images were then processed with Imaris software (Bitplane). Supplemental videos created from these image stacks are maximum intensity projections processed in ImageJ (NIH) and affined with StackReg plugin [29]. Cell tracking and measurement of cell motility parameters were performed using the Imaris software (Bitplane). Only cells tracks longer than 600 seconds were analyzed.

### Isolation of T cell populations and sorting

To isolate pure populations of CD4^+^ or CD8^+^ T cells from reconstituted mice, spleen cell suspensions were first depleted for non-T cells. For this, a cocktail of biotinylated MoAbs from BDPharmingen was used: anti-CD11b; CD11c, TER119, CD19, B220, PDCA1. For CD4^+^ T cells purification an anti-CD8β Ab was also added to the cocktail of depleting Abs. For CD8^+^ T cells purification, an anti-CD4 Ab was added to the cocktail of depleting Abs. Streptavidin magnetic beads (Dynal, Invitrogen, Carlsbad, CA, USA) were used to remove non-T cells. Enriched cell suspensions were further labeled with anti-CD3, anti-CD4 or anti-CD8β MoAbs and Abs recognizing the TCR transgenes (Vβ6 for CD4^+^ T cells; and T3.70 for CD8^+^ T cells). Labeled CD4^+^ or CD8^+^ T cells were then sorted twice in FACSAria (Becton Dickinson, Franklin Lakes, NJ, USA). The CD4^+^ T cells were sorted based on their CD3^+^CD4^+^Vβ6^+^ phenotype; the CD8^+^ T cells were sorted based on their CD3^+^CD8β^+^DSRed^+^T3.70^+^ phenotype.

### Expression of MHC class II and antigen presentation by CD8^+^ T cells

Female Rag2^**-/-**^ mice were sub-lethally irradiated and injected with either female or male Rag2^**-/-**^GFP^+^ BM. Three days later they received Mo male-specific TCR Tg CD4^+^ and CD8^+^ T cells. At different time points after T cell transfer, spleen cell suspensions were incubated with the 2.4G2 Mo Ab and 2% mouse serum to block Fc receptor binding. Cells were labeled with anti-MHC class II I-A^b^ (M5.114.15.2, eBioscience) and IA^d^ (AMS-32.1, BD Pharmingen) and analyzed in a FACSCanto, or studied by confocal microscopy. DsRed CD8^+^ T cells were then sorted in a FACS Aria, and deposited on slides saturated with 0.1% poly-L-Lysine (Sigma-Aldrich, St.Louis, Missouri, USA). Cells were fixed with 2% PFA, and incubated with Image-IT FX signal enhancer (Molecular Probes, Eugene, Oregon, USA). Cells were then labeled with the above anti-MHC class II^b^ or II^d^ MoAb or isotype controls, washed and incubated with a goat anti-rat Ab conjugated to AF568 (Molecular Probes). Confocal microscopy was performed with the Zeiss LSM700 axioplan (Carl Zeiss Micro-Imaging GmbH, Germany) or Leica SP5 microscopes (Leica Microsystems GmbH, Wetzlar, Germany). For MHC class II staining on B cells, total splenocytes from B6 mice were deposited on the microscope slides and stained with anti-IgD coupled to FITC (11-26c.2a, BD Pharmigen), the biotinylated anti-MHC class II Abs (I-A^b^, M5.114.15.2, eBioscience and IA^d^, AMS-32.1, BD Pharmingen) and streptavidin AF633.

To study antigen presentation capacity, 2x10^5^ double-sorted naïve Marilyn Mo TCR-Tg cells were labeled with CellTrace Violet, and incubated with 4x10^5^ double-sorted monoclonal HY specific CD8^+^ T cells. To isolate pure populations of CD4^+^ and CD8^+^ T cells, spleen cell suspensions were first depleted of non-T cells by magnetic sorting and each T cell population was labeled with four Mo Abs: anti-CD3ε, anti-CD4 or CD8β and Abs recognizing the TCR Tg. Debris and doublets were removed by gating, and CD4^+^ or CD8β^+^ populations were sorted twice using FACSAria (Beckton-Dickinson, USA). Sorted CD4^+^ and CD8^+^ T cells were mixed in 1:2 ratio, centrifuged at 200g for 2 minutes to accelerate CD4^+^/CD8^+^ contacts and, when mentioned, 1μg/ml of Marilyn or the HY peptide recognized by CD8^+^ TCR-Tg cells and presented by MHC class I were added. Cell division was evaluated by the dilution of the CellTrace Violet in day 4 cultures.

### Analysis of the functional properties of CD4^+^ T cells

In each experiment the CD4^+^ T cells were sorted from spleen cell suspensions pooled from 5 (day 3 after T cell transfer) and 3 (day 7 after T cell transfer) mice, as described above. The total RNA was isolated using RNeasy Mini or Micro kits (Qiagen, Corp., Venlo, The Netherlands), and gene expression analysis was performed the using mouse T Cell Anergy and Immune Tolerance PCR Array (Qiagen, Corp., Venlo, The Netherlands) according to the manufacturer’s instructions. Data analysis was performed using the Excel-based PCR Array Data Analysis Template provided. Expression was normalized to housekeeping genes, quantified according to the 2 –^ΔΔCt^ method and expressed as fold change. The Student t-test was used to evaluate significant differences. Statistically significant differentially expressed genes were further validated by real-time PCR using primer sequences designed with Primer3 software (http://primer3.ut.ee/) and the same RNA samples previously used for PCR arrays. RNA was first amplified using MessageAmpII aRNA amplification kit (Ambion^®^, Life Technologies, Carlsbad, California, USA) and reverse-transcribed using random hexamers (Applied Biosystems, Foster City, California, USA). Data analysis was performed as described above.

## Results

### Experimental strategy

This study aimed to identify the first interactions between naïve T cells and DCs. Since circulating T lymphocytes injected i.v. migrate to the spleen before reaching the LNs, these first T/Ag^+^DC contacts should be studied in the spleen. Therefore, we developed methods allowing TPLSM imaging in the spleen.

**Fig 1:**
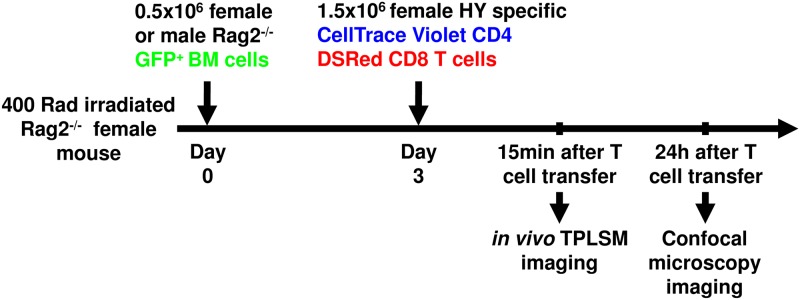
Experimental strategy used to evaluate the first T/DC interactions in the spleen. The panel shows the kinetics of injection, the number and color labeling of each cell type, and the time points when experimental data was collected. Lineage^-^ BM cells (0.5×10^6^) from male or female Rag2^-/-^ mice expressing GPF under chicken beta-actin promoter were injected i.v. in sub-lethally irradiated 400Rad (lethal dose 1300Rad) Rag2^-/-^ hosts. Three days later, recipients were adoptively transferred with 1.5×10^6^ monoclonal TCR-Tg T cells specific for the male antigen: Cell Trace Violet labeled Marilyn CD4^+^ T cells and DSRed^+^ CD8^+^ T cells.

We used Rag2^-/-^ female mice as hosts, which were sub-lethally irradiated (400Rad) and injected i.v. with male or female Lin^-^ bone marrow (BM) cells from Rag2^-/-^ mice expressing GFP under chicken beta actin promoter (Rag2^-/-^ GFP^+^ BM). Three to 3.5 days after BM injection these hosts were injected with 1.5x10^6^ naïve Mo TCR-Tg CD4^+^ (blue) and CD8^+^ T cells (red) specific for the male antigen (Fig 1).

**Fig 2:**
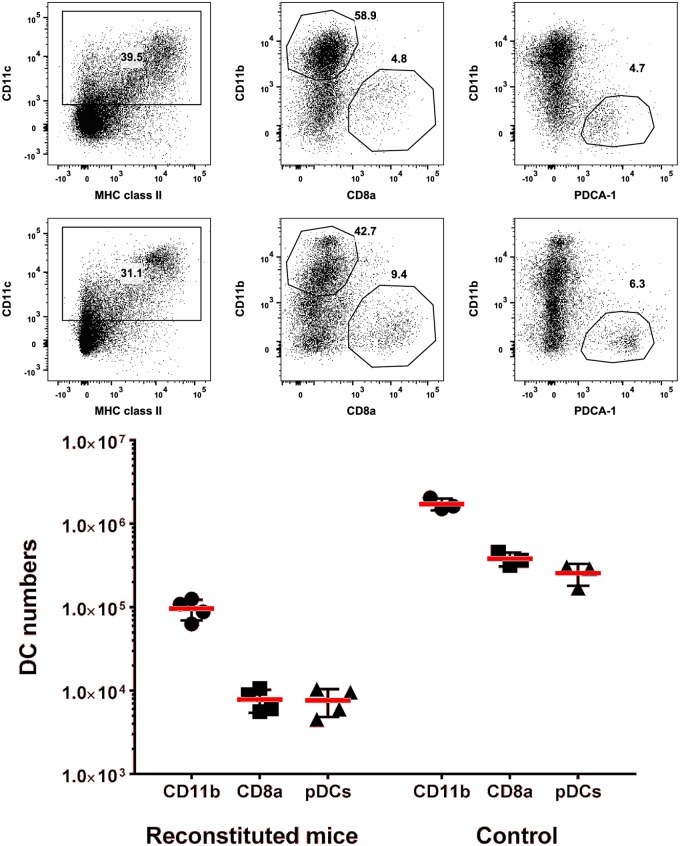
Characterization of BM derived cells in the spleen of mice injected with Rag2^-/-^GFP^+^ BM. **Upper panels**: Rag2^-/-^ female mice were irradiated (400Rad) and injected i.v. with 0.5×10^6^ male Rag2^**-/-**^ GFP^+^ BM cells. Results show the phenotype and frequencies of GFP^+^ cells in the spleen, 3.5 days after BM transfer; **Middle panels**: the same populations in a control Rag2^**-/-**^ female mouse, studied simultaneously. Results are from one mouse representative of 4 mice studied in two independent experiments. The gate to identify CD11c^+^ and MHC class II expression was established in non-labeled cells. The MFI of MHC class II expression by CD11c^+^ cells for reconstituted mice was 9131±4055 (n = 4), and for Rag2^-/-^ mice 10299±4547 (n = 3) (not statistically significant). **Lower panels**: the numbers of the different DC populations in reconstituted and control mice. Symbols show individual mice and bars the mean±SD of all the mice studied. **Please note**: Although the numbers of DCs were much lower in BM reconstituted/T cell injected animals than in non irradiated mice, the number of T cells injected was also proportionally much lower than that present in an intact spleen.

This system was required for the visualization of the first T cell/Ag^+^DC interactions in the intact spleen, for the following reasons: (i) the small spleen size of the Rag2^-/-^ hosts allowed performing TPLSM in the spleen, which was not possible in normal mice; (ii) the light irradiation of host mice was required to allow the reconstitution by the injected Rag2^-/-^ GFP^+^ BM. In normal mice, the DCs are continuously renewed from BM precursors [30], migrating from the blood through the red pulp (RP). Since DC subsets segregate properly in the spleens of Rag deficient hosts [31], the injected Rag2^-/-^ GFP^+^ BM should continuously export non-manipulated GFP^+^ DCs that reach the spleen by 3.5 days after BM injection, aiming to approach the conditions of DC renewal from the BM precursors in normal mice; (iii) the use of Rag2^-/-^ GFP^+^ BM for reconstitution ensured that GFP^+^ T or B cells were not generated. It was described that the injection of the Rag^-/-^ BM reconstitutes the normal DC populations, which are not affected in their differentiation or functions [31, 32]. Indeed, by 3.5 days after the injection of Rag2^-/-^ GFP^+^ BM, the frequencies of DC subsets in reconstituted spleens resembled that found in Rag^-/-^ hosts (Fig 2). Only CD8α^+^ DCs were less abundant, but their frequency increased later on (not shown). Remaining GFP^+^ cells were immature myeloid lineage cells, expressing Mac-1 but failing to express MHC class II (not shown).

(iv) the irradiation of the host mice was also required to eliminate the majority of the non-labeled endogenous DCs; thus, the first T/DC interactions should occur between the injected T cells and the DCs generated from the injected Rag2^-/-^ GFP^+^ BM; (v) to guarantee the visualization of the first cognate T/DC interactions, we had to exclude that the injected T cells would have previously interacted with non-labeled DCs from the host cross-presenting either the nominal Ag, or other cross-reactive Ags. Therefore, we had to use the male-specific TCR transgenic cells system. Both Monoclonal (Mo) TCR-Tg CD4^+^ and Mo TCR-Tg CD8^+^ T cells that recognize different peptides of the same male antigen are available. Importantly, the male antigen model is the only TCR-Tg system where the T cells are not cross-reactive [4, 33], nor stimulated by cross-presentation [1]. These TCR-Tg cells thus differ from all other TCR-Tg models, in particular from the OT-I Tg mice, in which T cells are extensively cross-reactive and have an important population of CD44^high^ cells even in Mo TCR-Tg “naïve” mice that were not immunized with OVA [33]. Since the male antigen is endogenous and continuously expressed by the DCs, this model addresses events occurring during a direct Ag presentation. It approaches the events that occur during the LCMV infection [16], where viral antigens expressed by the infected DCs are directly presented to Ag-specific T cells. It differs from previous systems, which used proteins, peptides [6, 8, 9] or non-replicating viruses for priming [13, 14]. The peptide shedding and cross-presentation occurring in other systems may hinder the identification of DCs that are presenting the Ag at a particular time point. Antigen concentrations may also decline faster, what may shorten the periods available for T/Ag^+^DC interactions. Lastly, to clearly visualize the composition and interactions within T/DC complexes the number of labeled antigen-specific cells T cells injected was lower than that usually used for TPLSM. In particular, we injected 1.5x10^6^ CD4^+^ and CD8^+^ T cells per mouse, whereas other studies used from 3-4x10^6^ [6, 7, 9, 13] to up to 1.5x10^7^ T cells per mouse [15, 21]. The latter numbers approach the total number of CD8^+^ T cells present in B6 mice (2x10^7^) [34]. The injection of low numbers of T and DC precursors was necessary to clearly visualize cell interactions. When higher numbers were injected, large cellular aggregates were formed and the discrimination of individual cells and their interaction patterns were no longer accurate. The presence of these aggregates could be one of the reasons why *in vivo* CD4^+^/CD8^+^ interactions were not evaluated previously.

Our system, however, may have limitations. We cannot formally exclude that the use of Rag2^-/-^ host mice, as well as the cell apoptosis occurring after irradiation may distort the behavior of the injected cells. However, since apoptotic cells are rapidly cleared *in vivo*, at the time points we studied (from 3.5 to 4.5 days after irradiation) we could not visualize any apoptotic cells. We also could not detect important modifications of the spleen stromal architecture. We could identify the different spleen compartments: the marginal zone (MZ), the white pulp (WP) with the central arteriole (CA) and the red pulp (RP) (S2 Fig).

### Motility behavior of individual cell types

**Fig 3:**
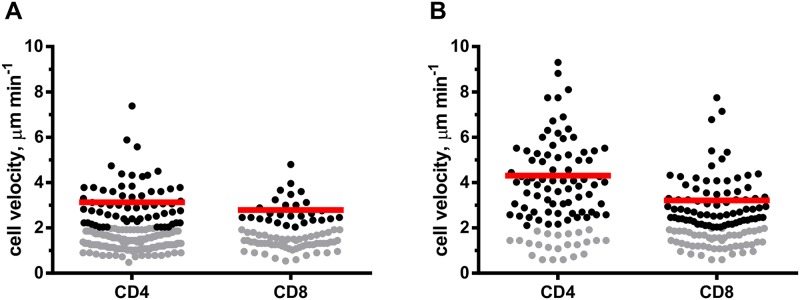
T cells migration velocities in the intact spleen. Rag2^**-/-**^ female mice were irradiated (400Rad) and injected i.v. with 0.5x10^6^ male (A) or female (B) Rag2^-/-^ GFP^+^ BM cells. 3.5 days later, 1.5x10^6^ of monoclonal male-specific TCR-Tg T cells were injected i.v.: CellTrace Violet labeled CD4^+^ T cells (blue), and DSRed CD8^+^ T cells (red). Each dot corresponds to an individual cell. Black dots show motile and grey dots represent arrested T cells (velocity <2 μm min^-1^). The red lines indicate the mean velocity values of motile T cells. **(A)** T cell motility was evaluated from 15 min to 4 hours after T cell transfer. A total of 198 CD4^+^ and 86 CD8^+^ T cell tracks ≥ 600 seconds were analyzed in three independent experiments. For CD4^+^ T cells the average track duration was 36.5 min, the average track length = 69.9 μm the average track displacement length = 15.2 μm. For CD8^+^ T cells the average track duration was 33.6 min, the average track length = 59.3 μm the average track displacement length = 12.4 μm. The velocity of CD4^+^ and CD8^+^ T cells was not statistically different (Student *t*-test). **(B)** Mice were studied 24h after T cell transfer in three independent experiments. Cell motility was evaluated as described above. For 100 CD4^+^ T cells the average track duration was 17.59 min, the average track length = 60.71μm the average track displacement length = 17.92 μm. For 125 CD8^+^ T cells the average track duration was 20.87 min, the average track length = 44.54 μm the average track displacement length = 13.93 μm.

After intravenous injection, T lymphocytes must cross the capillary networks of the liver and lungs before reaching the spleen, this traffic being more rapid for CD4^+^ than for CD8^+^ T cells [35]. Thus, from 15 minutes to 4 hours after T cell transfer T lymphocytes were rarely found in the spleen, were all localized in the red pulp and T/DC complexes were not detected. Therefore, at these time-points we could study the motility of individual T cells in the putative absence of previous T cell/DC contacts (S1 Video). The velocity of individual cells was not constant. The migration of DCs was in a stop-and-go pattern (S2 Video). In a similar way, a slow moving T cell could suddenly increase and further decrease its velocity along the cell track (S1 Video). A total of 198 CD4^+^ and 86 CD8^+^ T cells tracks were analyzed (Fig 3A).

The percentage of “arrested” CD4^+^ and CD8^+^ T cells (average velocity <2 μm min^-1^) was similar, (68.2% and 68.5% respectively) and comparable to that previously reported (~62%) [19]. Motile CD4^+^ T cells moved on average at 3.12±1.03 μm min^-1^, compared to 9–11 μm min^-1^ velocity reported in the LN [7, 21]. Motile CD8^+^ T cells in the spleen moved at 2.8±0.63 μm min^-1^, while in the LN CD8^+^ T cell average velocities were reported to range from 7.9 to 11.9 μm min^-1^ [8, 20].

Due to a large spleen size, T cell velocities in normal mice cannot be measured *in vivo* by TPLSM. The behavior of T cells is thus analyzed *ex vivo* in spleen tissue sections [22, 23]. It was found that in the absence of antigen TCR-Tg and WT T cells had overlapping behavior, that the presence of antigen reduced the velocity of antigen-specific cells, and that T cells in the spleen sections moved much slower than in the LN. As compared to our results the average velocities of T cells in these spleen sections were higher in the absence of antigen stimulation (average 5 μm min^-1^) or equivalent in the presence of Ag to those we report here (average 2.5–3 μm min^-1^).

To study if the reduced velocity of T cells in our system was due to the presence of Ag, we used the same experimental system where DCs were female, and thus Ag^+^ cells were absent (Fig 3B, S3 Video). In these conditions the number of arrested cells was reduced from 68% to 23% (CD4 T cells) and to 40% for CD8 T cells. The average velocity of CD4 T cells increased from 3.1 to 4.3±1.64 μm min^-1^ and for CD8 T cells from 2.8 to 3.2±1.17 μm min^-1^, i.e., were still slightly lower than the 5 μm min^-1^ reported in spleen sections in the absence of Ag.

The differences between previous and current measurements could be due to various factors: (i) we employed a slower frame rate to collect the images (1 image/min versus 2–6 images/min in the two other studies), since it smoothens the large fluctuations in instant velocities associated with T cell motility. However, this decreased frame rate was later shown to yield a lower mean velocity as compared to increased frame rates [22]. Therefore, our reduced frame rate may explain the reduced average velocity; (ii) the artificial experimental setting used in the study could also affect T cell motility rates. (a) The Rag2^-/-^ TCR-Tg T cells could have different motility as compared to WT T cells. This possibility is unlikely since the direct comparison of TCR-Tg and WT T cell behavior in the WT spleen of non-immunized mice showed that both populations had overlapping behavior and similar velocity averages; (b) The slower velocity of the T cells could be due to interactions with other (non-hematopoietic) cells, such as stromal cells, that were shown to express MHC II as well as MHC I [36]. This possibility is also unlikely since such stromal cells should also be present in the WT spleen. (c) The location of the T cells in particular splenic compartments and the time after T cell transfer when the velocity is studied could also have a major impact on the average T cell velocity. We studied T cells that had just entered the spleen and were thus located in the blood present in the red pulp. Others have studied much later time points (several days after T cell transfer) [22, 23], when T cells are mainly localized in the white pulp. The reduced velocity in the red pulp is to be expected, since the open structure of the splenic RP (where the cells are located in the blood) is different from the dense three-dimensional stromal network found in other zones of the spleen as well as in the LN. It was shown that in contrast to macrophages and granulocytes that crawl along flat surfaces, lymphocytes only move within dense three-dimensional networks [37]. Therefore, it is possible that our experimental system may impact T cell motility in the spleen but, if so, these modifications are not pronounced. The lower velocity of HY specific Rag2^-/-^ TCR Tg T cells in the Rag2^-/-^ spleen can be explained by the lower frame rate and the time points used to collect the images. It must be noted however, that the selection of these time points was required to analyze the first T/Ag^+^DC interactions.

### T/DC initial contacts and the formation of T/DC complexes

To guarantee the visualization of the first T/Ag^+^DC interactions, we studied the spleens of injected hosts from 4 (no T/DC complexes) to 7 hours after T cell transfer. During this time frame such interactions were very rare with no more than one-to-three events occurring in each TPLSM imaging experiment. All initial T/DC interactions occurred in the red pulp (*n* = 27) between a single lymphocyte and a single Ag^**+**^DC. It was proposed that within the first 24 hours after immunization all T/Ag^+^DC interactions were always transitory [20]. While all CD4^+^/Ag^+^DC interactions were indeed transitory (*t*<5min) (*n* = 11), all CD8^+^ T cells (*n* = 16) formed immediate stable interactions with Ag^+^DC (*t*>30min, S4 Video). Since our recording time spanned from 4 hours (when no interactions were found) to 7 hours after T cell transfer, it is very unlikely that CD8^+^ T cells ever formed transitory interactions with Ag^+^DC. The stable CD8^+^/Ag^+^DC interactions did not induce the arrest of CD8^+^ T cell or the Ag^**+**^DC. The CD8^+^ T lymphocytes rolled at the DC surface, both cells migrating as a complex (S4 Video).

Some of these stable CD8^+^/Ag^+^DC complexes already evolved to form ternary (CD4^+^/CD8^+^/Ag^+^DC) structures during this imaging period. These ternary complexes were always initiated by a stable CD8^+^/Ag^+^DC contact but could be of two types.

**Fig 4:**
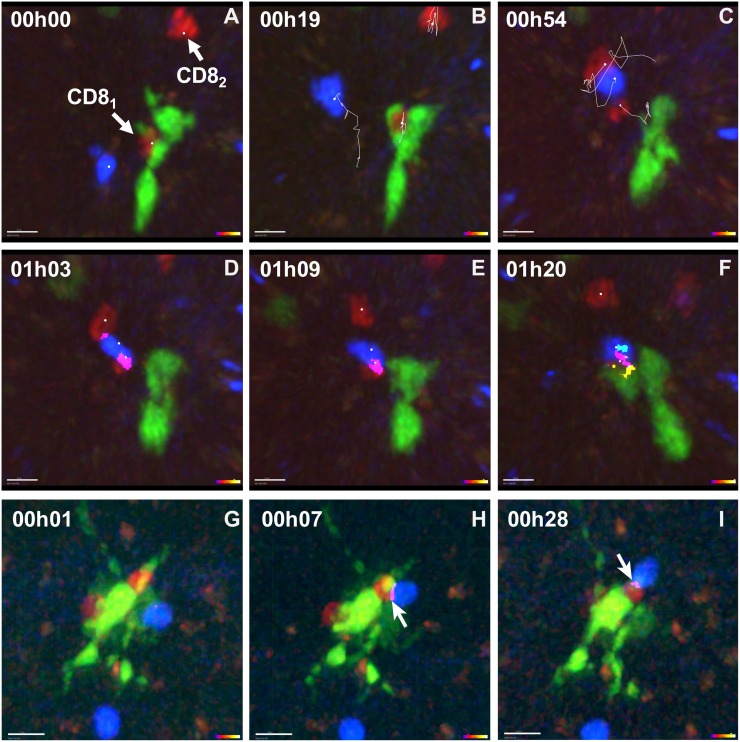
The formation of ternary T/Ag^+^DC complexes. Rag2^**-/-**^ female mice were irradiated (400Rad), reconstituted with Ag^+^ (male) Rag2^-/-^GFP^+^ BM cells and 3.5 days later received Mo TCR Tg CD4^+^ (blue) and CD8^+^ (red) T cells as described in Fig 1. TPLSM imaging of the intact spleen was performed from 4–7 hours after T cell transfer. Results show the two different types of complexes and are from two independent experiments out of the three performed. **(A-F): 1st type**: snap-shots of the TPLSM imaging from S5 Video; **(A)** initial cell locations; **(B-C)** the tracks of the different cells: **(B)** from 0 until 19min; **(C)** from 34 until 54min; **(D-F)** cellular interactions at the indicated time points: between a CD8^+^ and a CD4^+^ T cell (**magenta)**; between CD8^+^ and the Ag^**+**^DC (**yellow**); between CD4^+^ and the Ag^**+**^DCs (**cyan)**. (**G-I): 2nd type**: snap-shots of the TPLSM imaging from S6 Video. **(G-I)** cellular interactions between CD8^+^ and a CD4^+^ T cells at the indicated time points, represented by the same colors as in (**D-F**). Arrows indicate CD4^+^/CD8^+^ T cell contact zones. Scale bars in both experiments correspond to 10 μm.

In the first type, after forming a stable CD8^+^/Ag^+^DC contact, the CD8^+^ T cell detached from the Ag^+^DC and bound to a nearby motile CD4^+^. This CD4^+^/CD8^+^ complex was recruited to Ag^+^DC binding, forming a stable ternary complex (S5 Video; Fig 4A–4F).

In the first example when we started imaging a CD8^+^ T cell (CD8_1_) bound to the Ag^**+**^DC, this contact being maintained for 53 minutes (Fig 4A and 4B). This CD8_1_ then detached from the Ag^**+**^DC and bound to a nearby CD4^+^ T cell (Fig 4C). This CD4^+^ T cell was previously motile, and when studied in 3D did not show any binding to the Ag^**+**^DC even when followed during all the experiment in a larger imaging field. The CD4/CD8_1_ T cell complex was maintained without apparent Ag^**+**^DC binding for five minutes, when a second CD8^+^ T cell (CD8_2_) also interacted with the same CD4^+^ T cell (Fig 4C). We were able to determine that this CD8_2_ had not interacted with a DC previously. We could follow the migration of CD8_2_ from the beginning of imaging in a larger imaging field. During this period, CD8_2_ never left the large image field, was motile, and did not bind to any DC dendrite. The contact of this motile CD8_2_ with the CD4^+^ T cells was transitory (*t* = 16 min), the CD8_2_ moving away from the CD4/CD8_1_ complex (Fig 4D). In contrast, the CD4/CD8_1_ complex was stable, and migrated slowly back towards the initial Ag^+^DC (Fig 4F). Once in contact with this Ag^+^DC, the CD4/CD8_1/_/Ag^+^DC complex remained stable until end of the experiment. The total time of the direct CD4/CD8_1_ contact was measured to ~ 30 minutes, but could have lasted longer since imaging was interrupted. These results show that after CD8^+^/Ag^+^DC stable interactions, CD8^+^ T cells acquired the capacity to form stable complexes with motile CD4^+^ T lymphocytes (see CD8_1_ in Fig 4A–4F, S5 Video) and to recruit them to the Ag^+^DC (*n* = 7) forming ternary complexes later on. In contrast, motile CD8^+^ T cells (CD8_2_) that had not previously interacted with an Ag^+^DC only formed transient CD4^+^/CD8^+^ T cell contacts (see CD8_2_ in Fig 4 and S5 Video).

In the second type of complexes the CD8^+^ T cell forming the first stable contact with the Ag^+^DC did not necessarily detach from that Ag^+^DC to bind to a nearby CD4^+^ T cell. The CD8^+^ T cell could remain attached to the Ag^+^DC, and the motile CD4^+^ T cell could be recruited to CD8^+^ T cell binding. In Fig 4G–4I and S6 Video the CD4^+^ T cell binds the Ag^+^DC and 6 minutes later establishes a long-term contact with the CD8^+^ T cell (Fig 4H). This CD4^+^/CD8^+^ interaction lasted 28 min, until imaging was interrupted (Fig 4I).

### Cognate T/DC interactions promote the migration of the Ag^+^DC to the T cell zone

**Fig 5:**
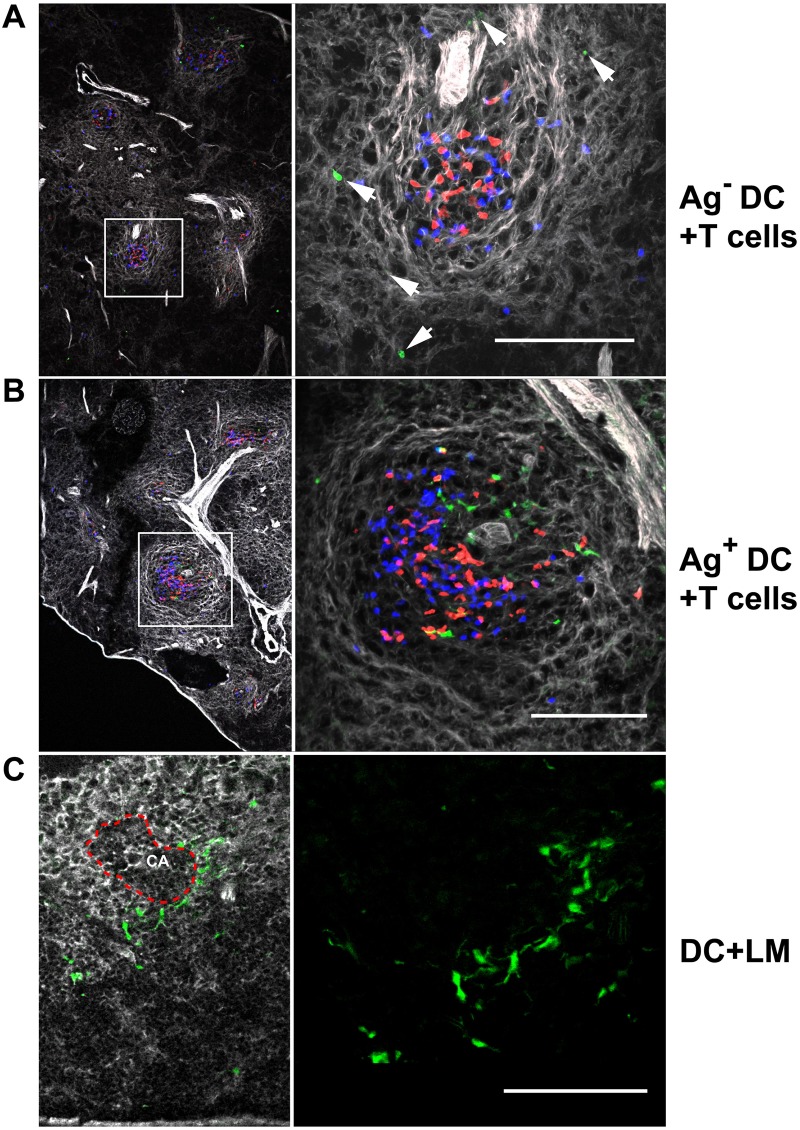
Cognate T/DC interactions induce DC activation and their migration to the spleen white pulp. Rag2^**-/-**^ female mice were irradiated (400Rad), and injected i.v. with female Ag^-^ (**A, C**) or male Ag^**+**^ **(B)** Rag2^-/-^GFP^+^ BM cells. Three days later mice were injected with 1.5x10^6^ male antigen-specific Mo TCR-Tg CD4^+^ (blue) and CD8^+^ (red) T cells (**A, B**); or 5,000 CFU of *Listeria monocytogenes* (LM) **(C)**. PFA fixed spleens were cryo-sectioned and studied by confocal microscope 24 hours after T cell injection (**A, B**); or 5 days after LM infection **(C)**. The spleen stroma was labeled with Alexa Fluor 635 phalloidin to better visualize the white pulp (grey). Arrows in **(A)** indicate the localization of DCs; the MZ in **(C)** is depicted by a red dashed line. Images are from a representative experiment out of five (**A, B**) and three **(C)** independent experiments. Scale bars correspond to 100 μm.

To evaluate a higher number of T/DC complexes, their localization and composition, we performed confocal imaging analysis of the spleens 24 hours after T cell transfer in the presence or the absence of the male antigen. At this time point, Ag^**+**^DCs were yet rare and CD4^+^ and CD8^+^ T cells were present in identical frequencies (53% and 47% of CD4^+^ and CD8^+^ T cells respectively). T cell/Ag^**+**^DCs complexes were of relative small size, allowing clear discrimination of individual cell/cell interactions by confocal microscopy. At later time points T cells and DCs formed large aggregates, where this discrimination was no longer accurate. We first assessed the localization of DCs and a possible influence of T cells on their localization. We found that female Ag^-^DCs did not form T/DC complexes and located outside the T cell zone either in the RP or in the MZ (Fig 5A).

In contrast, cognate T/DC interactions induced the activation and the migration of DCs to the spleen T cell zone (or periarteriolar lymphoid sheaths, PALS): The majority of male Ag^+^DCs located in the PALS, exhibited dendrite morphology, and formed multiple apparent contacts with T lymphocytes (Fig 5B). Typically, more than half of the total volume of Ag^+^DCs was deployed as dendrites, radiating on average 19.98 μm (SE = 1, *n* = 104) and in some cases ~50 μm away from the soma of the cell.

To evaluate whether the migration of DCs to the WP depends on their activation status or requires a T/DC contact, Rag2^-/-^ hosts, injected with Rag2^-/-^ GFP^+^ BM, were infected with *Listeria monocytogenes* (LM) and studied from 24 hours up to 5 days after infection. Infection with LM activated GFP^+^DCs and induced the formation of multiple dendrites, however these DCs accumulated in the MZ even when studied 5 days after infection (Fig 5C). These results indicate that DC activation alone was not sufficient to promote the migration of DCs to the white pulp and required cognate T/DC interactions.

In order to establish whether CD4^+^ or CD8^+^ T cells induced the migration of Ag^+^DCs to the T cell zone, we reconstituted Rag2^-/-^ mice with female or male Rag2^-/-^ GFP^+^ BM, and 3 days later with HY specific CD4^+^ and CD8^+^ T cells alone or together. The analysis of cell localization in the spleens 24 hours after T cell transfer confirmed that Ag^-^DCs do not migrate to the WP (S3A–S3C Fig). The migration of Ag^+^DC to the PALS was induced by either CD4^+^ or CD8^+^ T cells (S3D–S3F Fig). Taken together, these results contrast with previous reports, claiming that innate *in vitro* stimulation with LPS or *in vivo* stimulation with adjuvants was sufficient to induce the migration of the DCs to the LN’s WP [6, 20]. It must be noted, however, that these previous experiments cannot exclude a role of T cells in DC migration. These experiments were performed in intact mice, where unlabeled host endogenous T cells were present and could promote DC migration to the WP.

### Cellular composition and interactions in T/Ag^+^DC complexes

**Fig 6:**
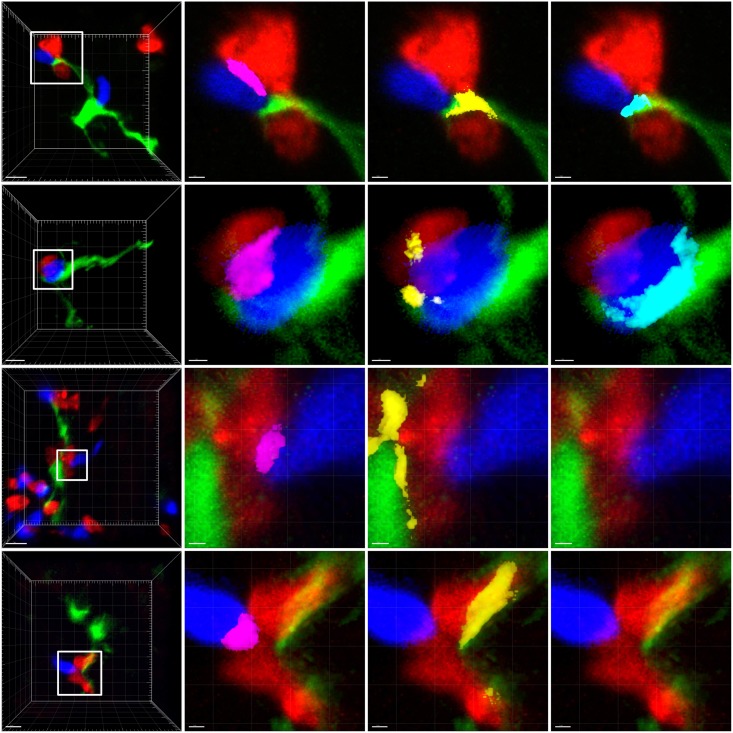
Evaluation of cellular interactions in T/Ag^+^DC complexes. Rag2^**-/-**^ female mice were irradiated (400Rad), injected i.v. with male Rag2^-/-^GFP^+^ BM (green) and three days later with male specific Mo TCR Tg: CD8^+^ (red) and CD4^+^ (blue) T cells, as described in Fig 1. 24 hours after T cell transfer spleens were cryo-sectioned and analyzed using confocal microscopy to identify the types of T/DC complexes. **Left panels**: The DC was first identified using a low magnification and the green channel alone. A number was attributed to each DC. Attached T cells were then visualized. The square delineates where T cells were present, selected for magnification (scale bar-5 μm). **Right panels**: The region of the DC where T cells were present was magnified (scale bar 1 μm). From left to right: The contacts between different cells: CD4^+^/CD8^+^: **Magenta**; CD8^+^/Ag^**+**^DCs: **Yellow**; CD4^+^/Ag^**+**^DCs: **Cyan**. The two upper panels show examples of complexes harboring CD4^+^ and CD8^+^ T cells interacting directly and also both interacting with the Ag^**+**^DCs. The two lower panels exemplify complexes where CD4^+^ and CD8^+^ interact directly, but only the CD8^+^ interacts with the Ag^**+**^DCs.

To better characterize the composition and cell-to-cell interaction patterns in the T cell/Ag^+^DC complexes, cellular aggregates were analyzed by confocal microscopy 24 hours after T cell transfer. We chose this strategy since it allowed us to section and study the whole spleen, and thus identify a much higher number of T/Ag^+^DC complexes than *in vivo* TPLSM imaging. Although these confocal studies are necessarily time snapshots, they provide complementary information about the frequency of different interaction types (S1 Note). The strategy used to identify cellular interaction patterns in T/Ag^+^DC complexes and examples of interactions are shown in Fig 6.

A total of seventy-six T cell/Ag^+^DC aggregates were analyzed in five independent experiments (Table 1).

**Table 1:** Cellular composition and interactions in T/Ag^+^DC complexes.

		Binary	CD8/DC	CD4/DC	Total binary	Ternary	4/DC/8	4/8/DC	Total ternary
Exp1	#DC:15		4	1	5		3	7	10
Exp2	#DCs:15		6	1	7		1	7	8
Exp3	#DCs:15		5	2	7		1	7	8
Exp4	#DCs:15		4	0	4		3	8	11
Exp5	#DCs:16		3	1	4		1	11	12
**Total**	**#DCs:76**		**22**	**5**	**27**		**9**	**40**	**49**
	%DCs		29	7	35		12	53	65
		**% of binary**	**83% p<0.01**			**% of ternary**		**82% p = 0.0001**	

Mice were injected with male Rag2^-/-^GFP^+^ BM and male-specific CD4^+^ and CD8^+^ T cells, as described in Fig 1. 24 hours after T cell transfer, cryo-sections of the spleens were studied by confocal microscopy. Cellular interactions were evaluated as described in Fig 6. Results are from five independent experiments.

27 of these complexes were binary, i.e., formed between a single T cell type and a single APC (35%). The majority of binary complexes had only one T cell and located in the red pulp, suggesting a recent generation. Within these binary complexes 83% were formed between a CD8^+^ T cell and the Ag^+^DC (Table 1). Since the frequency of CD8^+^ T cells (47%) was similar to that of CD4^+^ T cells (53%) 24 hours after T cell transfer, CD4^+^ and CD8^+^ T cells should have a similar probability to bind an Ag^+^DC. The significantly higher frequency of CD8^+^/Ag^+^DC versus CD4^+^/Ag^+^DC complexes (p<0.01) likely reflects the capacity of CD8^+^ T cells to form immediate stable contacts with the Ag^+^DC, as it was observed in our TPLSM imaging studies. Since our TPLSM imaging showed that CD4^+^/Ag^+^DC interactions were always of short duration, CD4^+^/Ag^+^DC complexes should have a much lower probability to be visualized by confocal snapshot imaging. 65% of the T/DC complexes were ternary. CD4^+^ and CD8^+^ T cells could bind independently to the Ag^**+**^DC, but such complexes were rare (*n* = 9). In 82% of ternary complexes (*n* = 40) CD4^+^ and CD8^+^ T cells also interacted directly with each other (p = 0.0001) **(**Table 1, Fig 6 **two upper panels)**. We also found another type of ternary complexes. In 22 DCs (17%) CD4^+^/CD8^+^ complexes were present in which only the CD8^+^ T cell interacted with the Ag^+^DC. **(**Fig 6, **two lower panels)**. In contrast, CD4^+^ T cells interacting with an Ag^**+**^DCs and a CD8^+^ lymphocyte with no apparent Ag^**+**^DCs binding were not detected.

Taken together, the study of CD4^+^ and CD8^+^ T cell behavior from four to seven hours after cell transfer showed that after a stable CD8^+^ T cell /Ag^+^DC interaction the CD8^+^ T cells could bind directly a motile CD4^+^ T cell, and recruit that cell to form a ternary CD4^+^/CD8^+^/Ag^+^DC complex (Fig 4). At 24 hours after T cell transfer, the majority (82%) of T cell/Ag^+^DC complexes were ternary, harboring CD4^+^ lymphocytes interacting directly with CD8^+^ T cells (Table 1).

### CD4^+^/CD8^+^ T cells interactions: Mechanisms and consequences

**Fig 7:**
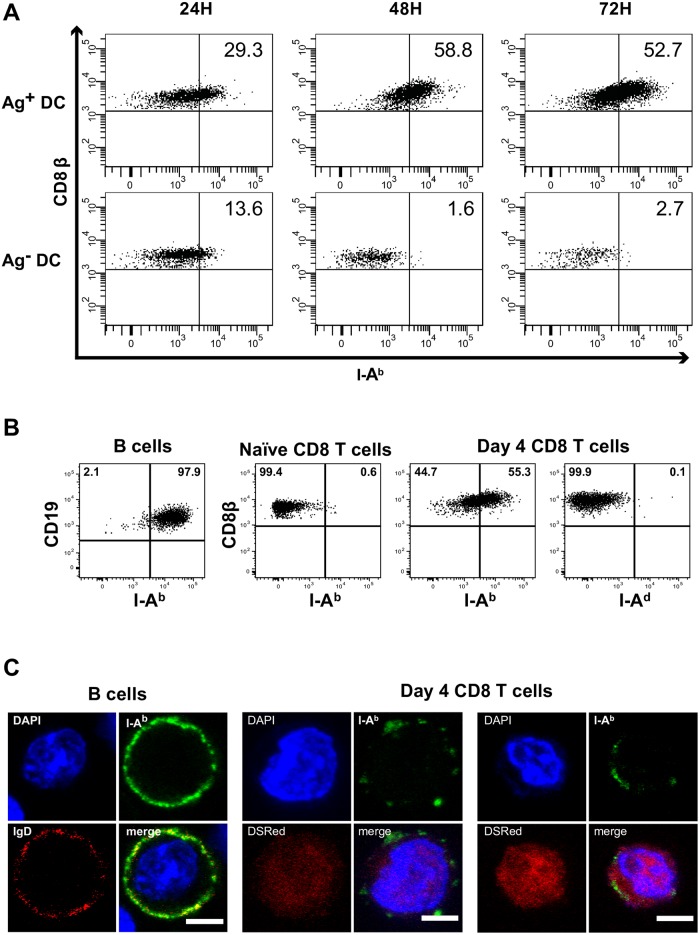
Expression of MHC class II by CD8^+^ T cells. Rag2^**-/-**^ female mice were irradiated (400Rad), injected i.v. with either Ag^+^ (male) BM or Ag^-^ (female) BM from Rag2^-/-^ donors, and male-specific Mo TCR-Tg CD4^+^ and CD8^+^ T cells as described in Fig 1. Results are from one representative experiment out of five performed and show: **(A)** The expression of the MHC class II^b^ by CD8^+^ T cells at different days after T cell transfer in mice injected with male (Ag^+^) Rag2^-/-^ GFP^+^ BM (Upper graphs) or female (Ag^-^) Rag2^-/-^ GFP^+^ BM (Lower graphs). **(B)** The specificity of MHC class II labeling: from left to right: expression of the MHC II^b^ in B cells, naïve and primed CD8^+^ T cells 4 days after T cell transfer; expression of the Balb/c class II^d^ in primed CD8^+^ T cells, 4 days after T cell transfer. **(C)** The distribution of MHC class II in B cells (left panels) and CD44^+^CD8^+^ T cells (middle and right panels). B lymphocytes were labeled with anti-IgD (red) and MHC class II Abs (green). The nuclei were stained with DAPI (blue). Male specific DSRed CD44^+^CD8^+^T cells were sorted from the spleen of female mice injected with male Rag2^-/-^ GFP^+^ BM and T cells 4 days after T cell transfer. Cells were labeled with anti-MHC class II^b^ or anti-MHC II^d^ (green), nuclei stained with DAPI (blue), and studied by confocal microscopy. Results show examples of MHC class II^b^ expression by two CD8^+^ lymphocytes (red). All CD8^+^ T cells showed similar patterns of MHC class II labeling, and none expressed MHC class II^d^ (not shown). Scale bars correspond to 5 μm.

Since CD4^+^/CD8^+^ T cells direct interactions required a previous stable contact between the CD8^+^ lymphocyte and the Ag^+^DC (Fig 4), we studied if such contact led to the acquisition of DC’ membrane fragments by the CD8^+^ T through trogocytosis, as it was suggested to occur during LCMV infection in normal mice [16]. Trogocytosis is known to occur through cognate interactions, and leads to the acquisition by the T cell some of DC’ membrane fragments, rather than just the synapsis area. In particular, the transfer of co-stimulatory molecules was demonstrated to occur [17, 18]. Therefore, the presence of such DC membrane fragments at the surface of CD8^+^ T cells could lead to the expression of MHC class II and co-stimulatory molecules by the CD8^+^ T cells, subsequently allowing them to function as antigen-presenting cells (APC) to naïve CD4^+^ T cells [14]. Trogocytosis indeed occurred in this system. CD44^+^CD8^+^ T cells that have been in contact with an Ag^**+**^DC expressed MHC class II IA^b^. In contrast, naïve CD8^+^ T cells, or CD8^+^ T cells adoptively transferred to mice receiving female BM did not express MHC class II (Fig 7).

This expression was not due to non-specific Ab binding. We blocked the Fc receptors previously to Ab labeling and these CD8^+^ T cells did not label with Abs recognizing the Balb/c MHC class II IA^d^ (Fig 7B). Moreover, confocal imaging of sorted CD44^+^CD8^+^ T cells showed that the MHC class II labeling distributed in patches at the CD8^+^ T cell surface (Fig 7C). This distribution pattern is consistent with acquisition of DC membranes by trogocytosis, while it differs from that found in cells expressing endogenous MHC class II (such as B cells), where surface labeling is homogeneously distributed (Fig 7C).

### Ag experienced CD8^+^ T cells can function as APCs to naïve CD4^+^ T cells

**Fig 8:**
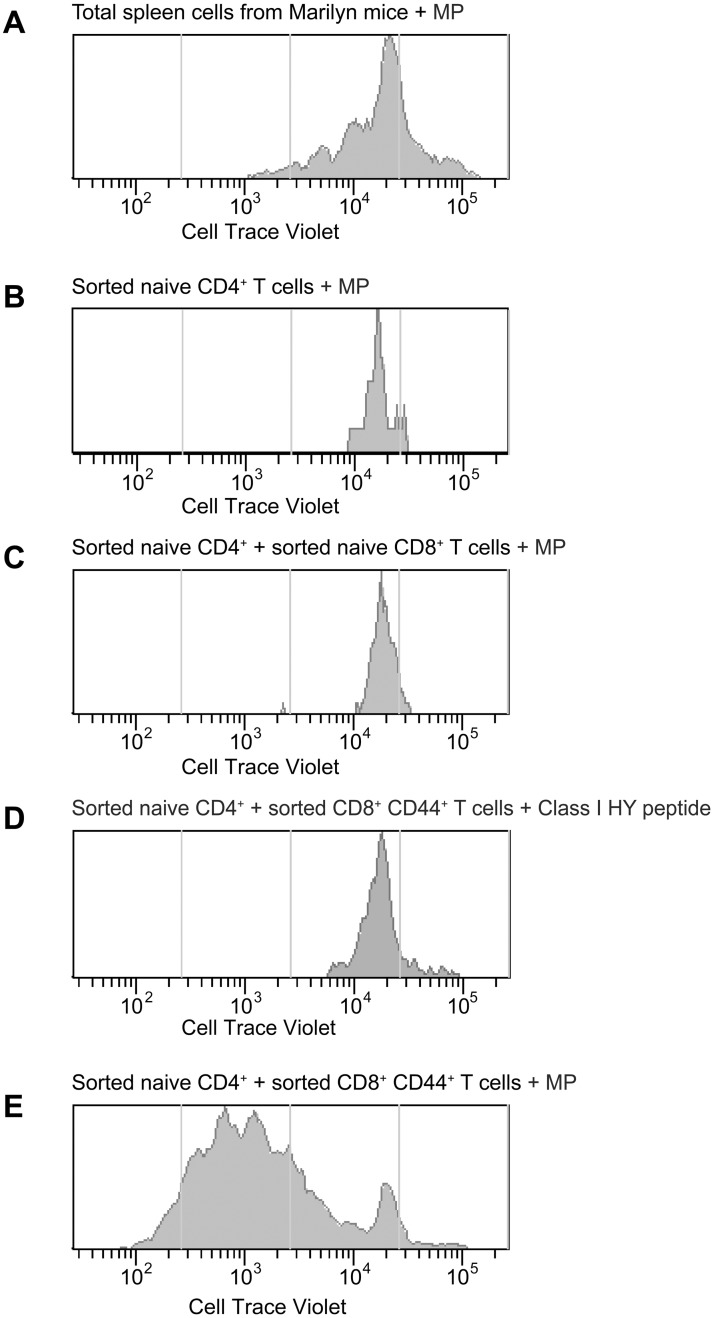
CD44^+^CD8^+^ T cells present the specific peptide and induce the division of naïve CD4^+^ T cells. Rag2^**-/-**^ female mice were irradiated (400Rad) and injected i.v. with either Ag^+^ (male) BM or Ag^-^ (female) Rag2^-/-^ GFP^+^ BM. Three days later they were injected i.v. with male-specific Mo TCR-Tg CD4^+^ and CD8^+^ T lymphocytes, as described in Fig 1. At day 4 after T cell transfer, CD8^+^ T cells were double-sorted from spleen cells suspensions pooled from 5–6 mice/group, and cultured in the presence of double-sorted CellTrace Violet labeled naïve CD4^+^ T cells from Marilyn mice and (when mentioned) the optimal concentration (1μg/ml) of Marilyn (MP) or the class I HY peptide. Results show CellTrace Violet dilution, as indicator of cell division in four days cultures. **(A)** Total spleen cells from Marilyn mice; **(B)** Double-sorted CD4^+^ T cells from Marilyn mice cultured alone; (**C)** Double-sorted CD4^+^ T cells from Marilyn mice cultured in the presence of double-sorted naïve CD8^+^ T cells recovered from mice injected with Ag^-^ (female) Rag2^-/-^ GFP^+^ BM; **(D)** Double-sorted CD4^+^ T cells from Marilyn mice cultured in the presence of double-sorted CD44^+^CD8^+^ T cells recovered from mice injected with Ag^+^ (male) Rag2^-/-^ GFP^+^ BM and 1μg/ml of the class I HY peptide recognized by the CD8^+^ TCR-Tg cells; **(E)** Double-sorted naïve CD4^+^ T cells cultured in the presence double-sorted CD44^+^CD8^+^ T cells from mice injected with male Rag2^-/-^ GFP^+^ BM and the MP.

We next wished to determine whether activated CD8^+^ T cells, by acquiring MHC class II and co-stimulatory molecules at the cell surface by trogocytosis, could become APCs to CD4^+^ T cells, i.e., would be able to present their specific peptide to naïve CD4^+^ T cells and induce their division. We had previously studied the optimal conditions of T cell stimulation with peptides or anti-CD3ε MoAbs. We found that a ratio of 2 DCs for 1 T cell was optimal (Tanchot, C. unpublished observations). To study the antigen presentation capacity of CD8^+^ MHC class II^+^ T cells, we reproduced these optimal conditions, replacing DCs with CD8^+^ T cells, i.e., a ratio of 2 CD8^+^ MHC class II^+^ T cells to 1 naïve CD4^+^ T cell. Thus, CD8^+^ T cells recovered from a pool of 5–6 mice injected with either female or male Rag2^-/-^ GFP^+^ BM were double-sorted 4 days after T cell transfer, and cultured in the presence of CellTrace Violet-labeled double-sorted naïve Marilyn Mo CD4^+^ T cells and the Marilyn specific peptide (MP). Division was evaluated by CellTrace Violet dilution (Fig 8).

To demonstrate the APC function of CD8^+^ T cells it was fundamental to exclude the presence of other APCs in these cultures: functional DCs, dead DCs or DCs fragments; it was also fundamental that CD4^+^ T cell division, if present, was strictly dependent on the supplementation of these cultures with the CD4^+^ T cell-specific Marilyn peptide (MP). Indeed, if CD44^+^CD8^+^ T cells could induce CD4^+^ T cell proliferation in the absence of MP, we could never exclude that this proliferation was induced by non-cognate CD4^+^/CD8^+^ T cell interactions and other bystander processes such as cytokines produced by the CD8^+^ T cells. All these aspects were addressed by the multiple controls shown in Fig 8.

Fig 8A shows that when total spleen cells from Marilyn mice were stimulated with MP, naïve CD4^+^ Marilyn T cells divided. This is our positive control, which showed that when DCs and the specific peptide were present, naïve CD4^+^ T cell division occurred. Fig 8B and 8C shows the controls for the efficiency of our purification procedures to remove DCs or DCs derived contaminants: double-sorted naïve Marilyn cells alone (**B**), or in the presence of double-sorted CD8^+^ T cells from mice injected with Ag^-^ female Rag2^-/-^ GFP^+^ BM **(C)** did not divide in the presence of the MP. Fig 8D excludes non-cognate CD4^+^/CD8^+^ interactions or/and cytokines secreted by CD8^+^ T cells as responsible for naïve CD4^+^ T cell proliferation. Thus MHC class II^+^ CD44^+^CD8^+^ T cells, double-sorted from mice immunized with male Rag2^-/-^ GFP^+^ BM, were put in contact with double sorted naïve Marilyn CD4^+^ naïve T cells. These cultures were supplemented with the HY peptide presented by MHC class I and recognized by the CD8^+^ T cells. Naïve CD4^+^ T cells also did not divide in these conditions, negating that non-cognate CD4^+^/CD8^+^ T cells interaction or/and cytokine secretion by CD8^+^ T cells could induce the division naïve CD4^+^ T cells. Fig 8E actually shows the experimental group. Double-sorted naïve CD4^+^ T cells divided extensively when put in contact with MHC class II^+^ CD44^+^CD8^+^ T cells and the MP.

These results directly demonstrate that only the CD8^+^ T cells that were in contact and trogocytosed Ag^+^DC membranes were able to present the Marilyn peptide to naïve CD4^+^ T cells, i.e., were able to function as APC to antigen-specific naïve CD4^+^ T cells. This result confirms the current accepted notion that naïve CD4^+^ T cell division depends of the recognition of a specific peptide presented by MHC class II, and co-stimulation. Indeed, only MHC class II presents the MP, and trogocytosis is known to lead to the expression of MHC class II and co-stimulatory molecules [17, 18].

We have to highlight why it was important to supplement T cell cultures with the MP rather than incubate CD8^+^ CD44^+^ MHC class II^+^ T cells with naïve CD4^+^ T cells directly. The purification procedures used to eliminate all contaminating DCs take a long time to perform. Since the peptide half-life in immature DCs is about 2.6 hours [38], the MP could not be present at the surface of CD8^+^ CD44^+^ MHC class II^+^ T cells after the procedures used for their purification, which took much longer than 2.6 hours to perform. Importantly, supplementation with the MP was required (i) to validate our purification methods; (ii) to demonstrate directly the APC function of CD8^+^ MHC class II^+^ T cells. Indeed, if MHC class II^+^ CD8^+^ T cells would induce naïve CD4^+^ T cell division in the absence of MP supplementation, we could never exclude that CD4^+^ division was induced by non-cognate CD4^+^/CD8^+^ interactions or/and cytokine secretion by activated CD8^+^ T cells. The APC function of the CD8^+^ T cells is only demonstrated because naïve CD4^+^ T cell divide in the presence of CD8^+^ CD44^+^ MHC class II^+^ T cells supplemented with the MP.

These results indicate that CD8^+^ T cells forming stable contacts with Ag^**+**^DCs were able to present the MP to naïve Marilyn CD4^+^ TCR-Tg T cells and induce their extensive division. This proliferation requires MP presentation and cannot rely only on IL-2 production since IL-2 alone can induce the proliferation of previously activated T cells but fails to induce the proliferation of naïve T cells [39, 40]. Therefore, these results demonstrate that after trogocytosis of DC membranes, CD8^+^ T cells also acquire the capacity to present the Ag and induce the extensive division of naïve CD4^+^ T cells. It must be noted that these MHC class II^+^ CD8^+^ T cells cannot be considered as professional APCs. Professional APCs have multiple additional properties including a fundamental role in Ag processing, likely not shared by these CD8^+^ T cells.

### The role of CD8^+^ T cells in the CD4^+^ T cells *in vivo* immune responses

The above results suggest that CD8^+^ T cells and their capacity to form immediate stable interactions with Ag^+^DC could influence *in vivo* CD4^+^ T cell immune responses by several concurring mechanisms: by inducing the precocious activation of immature Ag^+^DC, allowing them to up-regulate the expression of MHC class II and thus present the specific peptide to naïve CD4^+^ T cells; by secreting cytokines and chemokines, which should modify the cytokine environment, modifying CD4^+^ migratory behavior and CD4^+^ differentiation; by directly binding motile naïve CD4^+^ T cells and recruiting them to Ag^+^DC binding; by directly activating naïve CD4^+^ T cells, inducing their division and differentiation into effector cells.

To evaluate whether CD8^+^ T cell help influenced CD4^+^ T cell responses, we studied the activation and the gene expression profiles of CD4^+^ T cells when immunized exclusively with Ag^**+**^DCs or also in the presence of CD8^+^ T lymphocytes.

**Fig 9:**
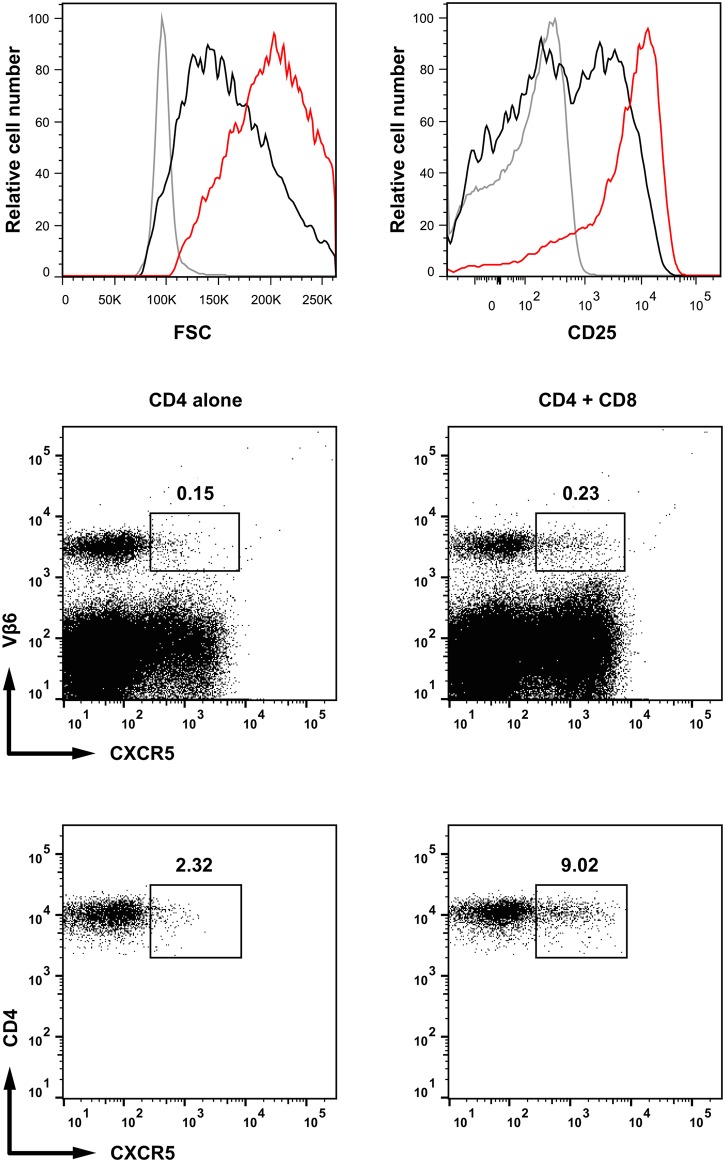
The role of CD8^+^ T cells in promoting CD4^+^ activation. Rag2^**-/-**^ female mice were irradiated (400Rad) and injected i.v. with Ag^+^ (male) Rag2^-/-^GFP^+^ BM cells. Three days later they received 1.5x10^6^ male-specific Mo TCR-Tg naïve Marilyn CD4^+^ T cells alone, or together with 1.5x10^6^ male specific Mo TCR-Tg CD8^+^ T cells. CD4^+^ T cells were studied three days after T cell transfer. **Upper histograms**: Cell size (left histograms) and CD25 expression (right histograms) of naïve CD4^+^ T cells (grey), CD4^+^ T cells injected alone (black) or together with CD8^+^ T cells (red). **Middle dot plots**: CXCR5 expression by male-specific Vβ6^+^ (CD4^+^) T cells injected alone (left) or together with male-specific CD8^+^ T cells (right). Numbers indicate the percentage of CXCR5 positive cells in the total spleen population. **Lower dot plots**: CXCR5 expression by CD4^+^ T cells gated on CD3^+^CD4^+^Vβ6^+^CD25^+^ from the middle dot plots. The gates defining positive cells were established in the same population not labeled with anti-CXCR5 Abs. Result are from one mouse representative of the four studied in two different experiments.

The presence of CD8^+^ T lymphocytes induced the early activation of CD4^+^ T cells, which showed significant increase in size and CD25 up-regulation, as well as CXCR5 expression (Fig 9).

These results demonstrate directly that the presence of CD8^+^ T cells induces the early activation of CD4^+^ T lymphocytes. Besides, CD4^+^ T cells gene expression profiles were modified (Table 2**)**.

**Table 2:** The role of CD8^+^ T cells in CD4^+^ responses.

	Day 3		Day 7	
Gene	Fold Change	*t*-TEST	Fold Change	*t*-TEST
	CD4+CD8/CD4	p-value	CD4+CD8/CD4	p-value
**effector molecules**				
*Ccl3*	**6.9**	0.01	**0.1**	0.004
*Gzmb*	**5.1**	0.003	**0.2**	0.01
*Il10*	**4.5**	0.0005	**0.1**	0.02
*Fasl*	**4.5**	0.02	n.s.	
*Prf1*	**4.0**	0.004	n.s.	
*Il5*	**3.5**	0.01	n.s.	
*Ifng*	**3.3**	0.05	n.s.	
*Il15*	n.s.		**2.5**	0.01
*Il2*	n.s.		**3.0**	0.01
**co-stimulators**				
*Icam1*	**2.6**	0.02	n.s.	
*Cd70*	**2.4**	0.03	n.s.	
*Il2ra*	**2.4**	0.02	n.s.	
*Cd40lg*	**2.2**	0.001	n.s.	
**activation markers**				
*Il10ra*	**2.8**	0.002	n.s.	
*Cdk4*	**2.1**	0.01	n.s.	
**transcriptional regulators**				
*Irf4*	**3.0**	0.02	n.s.	
*Jun*	**2.5**	0.02	n.s.	
*Fos*	**2.1**	0.04	n.s.	
**Tfh markers**				
*Cxcr5*	**4.2**	0.05	1.8	0.05
**other functions**				
*Cblb*	**2.0**	0.02	n.s.	

Rag2^**-/-**^ female mice were irradiated (400Rad) and injected i.v. with male Rag2^-/-^GFP^+^ BM cells. Three days later they received male-specific Mo TCR-Tg naïve CD4^+^ T cells alone, or together with CD8^+^ T cells as described in Fig 9. In each experiment, splenic CD4^+^ T cells were sorted from a pool of 5 mice at day three and 3 mice at day seven after T cell transfer. CD4^+^ T cells gene expression was studied using the mouse T Cell Anergy and Immune Tolerance PCR Array. Results show fold changes, and t-test p values from pools of five (day 3) and three (day 7) independent experiments. The p values were calculated based on a Student’s t-test of the replicate 2^–ΔCt^ values for each gene in the CD4/CD8 group and CD4 alone groups at day 3 and day 7 separately, according to the instructions for analyzing PCR Array results from Invitrogen.

Thus, CDK4 and CDK6 expressed during division were up regulated. The expression of multiple mRNAs coding for effector functions, co-stimulatory and activation markers and some transcription factors were also increased. This effect was more marked at the beginning of the immune responses, but *Il2* and *Il15* were yet up regulated at day seven after priming (Table 2). In three out of the 5 experiments performed, the number of CD4^+^ T cells recovered at day three after priming was also much higher (not shown). We conclude that the presence of CD8^+^ T cells modulates CD4^+^ T cell immune responses *in vivo*, promoting their early activation and differentiation.

## Discussion

To allow TPLSM imaging of the first T/DC interactions in the spleen we had to use an artificial Rag2^-/-^ system, where the small spleen size would allow such imaging. We used irradiated Rag2^-/-^ mice as hosts, injected with Lineage^-^ Rag2^-/-^ GFP^+^ BM, followed 3.5 days later by the injection of low numbers of T cells. This protocol was selected after multiple preliminary experiments, where we altered the numbers and kinetics of the cell injections. In these conditions the BM derived DCs do not have to compete with DCs resident in the spleen, and we could not find that the absence of this competition accelerated DC colonization of the Rag2^-/-^ spleen. BM derived DCs colonized the host spleen by 3.5 days after BM injection, as described in the studies of DC renewal in the spleen of normal mice [30]. We studied the spleen by 3.5 days after light irradiation and did not found major modifications of the spleen architecture or images of apoptotic cells. Moreover, the migration properties of transferred T cells mimicked those described for normal T cells in tissue sections of the normal spleen. Lastly, the adoptive transfer of naïve T cells to a lymphopenic host could induce their homeostatic proliferation (HP) [41]. The use of monoclonal T cells expressing TCR receptors specific for the male antigen excluded this possible caveat. The HY TCR Tg model is the sole system where Mo T cells are not cross-reactive and naïve cells do not undergo HP when transferred to lymphopenic hosts [33].

Our data has implications in the present concepts of T/DC and T/T interactions, their mechanisms and consequences. We show that the behavior of T cells and DCs, previously described in the LNs cannot be extrapolated to the spleen, which has a different architecture and is specialized in the clearing of blood-born pathogens (S2 Note). The major findings reported here are that the initial T/DC interactions are not necessarily of short duration; that CD8^+^ T cells bind directly to CD4^+^ cells; and that the vast majority of T/DC complexes are ternary, harboring both T/DC and CD4^+^/CD8^+^ direct interactions. Lastly, CD8^+^ T cells may function as APCs to CD4^+^ T cells, playing an important role in CD4 immune responses *in vitro* and *in vivo*.

Our data indicates that the “three-phase model” [20] (previously proposed to describe all initial T/DC interactions) does not apply to all T cells/immunization procedures. This model proposes that during the first 24 hours after T cell transfer, T/DC interactions were always of short duration [20]. Stable contacts leading to T cell activation would only be established later, being maintained until cell division, which would abrogate T/DC binding. In contrast, in the above experiments all the CD8^+^ T cells encountering an Ag^+^DC formed immediate stable contacts already at 4–7 hours after T cell transfer. This preference of CD8^+^ T cells rather than CD4^+^ T cells to form stable T/Ag^**+**^DC interactions likely reflects the relatively higher capacity of immature DCs (which express low levels of MHC class II) to present endogenous antigens via MHC class I. Antigen presentation by MHC class I is less dependent on DC maturation than antigen presentation by MHC class II [42, 43]. Although CD4^+^ T cells can also recognize endogenous antigens directly, this recognition requires previous DC activation and MHC class II up-regulation.

Overall, the formation of immediate stable of T/Ag^+^DC interactions most likely depends on the relative capacity of each DC to present that antigen [42, 43]. It is possible that in other immune reactions, where DCs are activated by innate stimulation and/or Ag is cross-presented, CD4^+^ rather than CD8^+^ T cells form immediate stable interactions with Ag^+^DCs. However, in all cases, the cells establishing immediate stable T/Ag^+^DC contacts will be strongly selected for early division and differentiation. Thus, immediate stable T/Ag^+^DC interactions reveal a major strategy to select the “best fit” T cells already at the earliest phase of the immune response.

We also show that stable CD8^+^ T cell/Ag^+^DC complexes have multiple repercussions in the initiation of the immune response. These interactions activate DCs which acquire dendrite morphology and migrate to the T cell zone. Notably, innate stimulation after infection with LM also induces DC activation, however these activated DCs failed to migrate to the T cell zone. These results contrast with previous reports where *in vitro* or *in vivo* innate signaling induced DC migration to LN’ PALS [6, 20]. Importantly, these experiments cannot discriminate if the migration of the DCs into the T cell zone was mediated by innate stimulation alone. These studies were performed in normal mice, where lymphocyte trapping is known to lead to the accumulation of all antigen-specific cells (dispersed through the body) in the LN draining the site of the infection [11]. Thus, in these experiments non-labeled endogenous antigen-specific T cells were very abundant, and may have contributed to the reported DC migration.

The formation of stable Ag^+^DC/CD8^+^ T cell contacts capacitate CD8^+^ T cells to bind naïve CD4^+^ T cells and to form stable CD4^+^/CD8^+^ complexes. These complexes together with Ag^+^DCs establish ternary complexes where CD4^+^ and CD8^+^ T cells are in close contact. Co-localization of CD4^+^ and CD8^+^ T cells was shown to depend of CCL3 and CCL4 chemokines [9]. We have shown that after LM-OVA infection, OT-I CD8^+^ T cells secrete these chemokines immediately after activation, preceding division, and in significantly higher amounts than the OT-II CD4^+^ T cells immunized simultaneously [10]. Such chemokine production by CD8^+^ T cells and activated Ag^+^DCs justifies the recruitment of nearby motile cells to the Ag^+^DCs but does not explain why the vast majority of T/Ag^+^DC complexes are ternary, i.e. harbor both CD4^+^ and CD8^+^ lymphocytes. The preferential formation of stable ternary complexes may be explained by two further events. Since DC maturation is required for efficient MHC class II antigen presentation, CD4^+^ T cells would only be able to form stable contacts with previously differentiated Ag^+^DC, i.e., with those DCs that had established a previous stable contact with a CD8^+^ T cell. Besides chemokine secretion, antigen-specific CD8^+^ T cells also have an alternative mechanism to recruit naïve antigen-specific CD4^+^ T cells. Upon Ag^**+**^DCs binding, CD8^+^ T cells acquire fragments of the DC membrane at their surface, becoming able to bind, present Ag, and activate naïve antigen-specific CD4^+^ lymphocytes. It must be noted that naïve T cell proliferation requires cognate interactions with Ag^+^DCs and co-stimulatory molecules, i.e., IL-2 alone cannot induce the division naïve cells [39, 40]. Therefore, the capacity of MHC class II^+^ CD8^+^ T cells to interact with and induce a peptide dependent division of naïve CD4^+^ T cells demonstrates that trogocytosis confers to CD8^+^ T cells an antigen-presenting capacity, currently believed to be restricted to Ag^+^DCs. The APC function of CD8^+^ T cells will likely have a particularly important role at the beginning of CD4^+^ T cell responses in accelerating CD4^+^ T cell activation. Indeed, this APC function of CD8^+^ T cells is precocious; it already occurs from four to seven hours after T cell transfer, when CD4^+^ T cells do not yet form stable direct CD4^+^/Ag^+^DC contacts [20]. Besides, the capacity of CD8^+^ T cells to function as APC should increase the number of APCs available, which is believed to be very limiting in early responses. Indeed, in natural infections, the initial number of DCs presenting the antigen is believed to be extremely low. As an example, 4 flu infectious particles in aerosols are sufficient to transmit the disease to 50% of the hosts [44]. Therefore, at the beginning of immune responses, when Ag^+^DCs are very rare and CD4^+^ T cells are unable to form stable CD4^+^/Ag^+^DC complexes, the capacity of CD8^+^ T cells to function as APC to CD4^+^ T lymphocytes could have a major impact in early CD4^+^ T cell activation and differentiation. Indeed, we found that the presence of antigen-specific CD8^+^ T lymphocytes modify the immune responses of antigen-specific CD4^+^ T cells *in vivo*. In particular, the presence of CD8^+^ T cells leads to early activation of the CD4^+^ T cells, as exemplified by their increased size, and significantly higher expression of CD25. Moreover, the presence of the CD8^+^ T cells increases the expression of mRNAs coding for multiple effector and adhesion molecules in the CD4^+^ T cells. The extent of this modulation requires further extensive characterization. We demonstrated that CD8^+^ T cells accelerate CD4 immune responses, but we could not discriminate if the modifications of CD4^+^ T cells’ gene expression profiles were solely due to different response kinetics, or depended on direct CD4^+^/CD8^+^ contacts. We could not detect the up-regulation of the expression of genes involved in TH2 or TH17 differentiation, but genes expressed by TH1 or TF_H_ were modified. Since only a fraction of CD4^+^ T cells expresses CXCR5, it is possible that at this early differentiation stage the CD4^+^ population is not yet committed to a single differentiation pathway, or that they are mixtures of TH1 and TF_H_. Also, it cannot be excluded that CD4^+^ T cells yet differentiate into another T_H_ subclass, specialized in help to CD8^+^ responses.

It must be considered if the data obtained in the present model can be extrapolated to events occurring during infection. Stimulation of CD8^+^ T cells by direct presentation is quite frequent, since pathogens frequently infect DCs. It occurs for example during the LCMV infection of normal mice. CD8^+^ T cells also interact with conventional DCs [16], and are able to present Ag and induce the division of CD4^+^ T cells. Therefore, the sequence of events we here describe is representative of at least some immune responses. Besides, the presence of direct CD4^+^/CD8^+^ interactions was also described in the *in vivo* response to ovalbumin [15].

The accumulating *in vivo* imaging reports reveal an unexpected heterogeneity and complexity of T cell behavior and T/DC interactions patterns. It is likely that the complex immune system modulate the intrinsic T and DC motility and migration capacities to adapt for optimal immune response to every individual challenge.

## Supporting information

S1 Fig Phenotype of naïve and activated CD4^+^ and CD8^+^ male-specific TCR Tg T cells. CD4^+^ Vβ6^+^ T cells (**upper panels**) and CD8^+^ T3.70^+^ T cells (**lower panels**) from male-specific TCR Tg mice were studied for CD25 and CD69 expression. **Most left dot plots** show the expression of CD25 in naïve T cells; **left dot plots** show the expression of CD25 in T cells on day 3 after transfer into Rag2^-/-^ hosts that were irradiated and reconstituted with male (Ag^+^) Rag2^-/-^ GFP^+^ BM cells; **right dot plots** show CD69 expression in naïve T cells from male-specific Tg mice; **most right dot plots** show CD69 expression in T cells on day 3 after transfer into Rag2^-/-^ hosts that were irradiated and reconstituted with male (Ag^+^) Rag2^-/-^ GFP^+^ BM cells.
(TIF)

S2 Fig Identification of the different anatomic structures of the spleen. Images show cross-sections of the spleen from Fig 5A. The spleen stroma is labeled with Alexa Fluor 635 phalloidin, CD4^+^ T cells labeled in blue and CD8^+^ T cells in red. **Upper panels**, left: phalloidin staining alone, arrows indicating the central arteriole (CA) of the white pulp (WP); right: the same cross-section showing labeled T cells accumulating in close proximity to the CA, defining the T cell zone or PALS. Scale bars corresponds to 100μm. **Lower Panels**, left: orange shading indicates the border of the WP and the red pulp (RP), and is defined as the marginal zone (MZ), square denotes the area magnified in right image; right: higher magnification view defining the white pulp (WP), central arteriole (CA), marginal zone (MZ) and red pulp (RP). Scale bars correspond to 100μ.
(TIF)

S3 Fig CD4^+^ and/or CD8^+^ T cells cognate T/DC interactions promote the migration of DCs to the spleen white pulp. Rag2^**-/-**^ female mice were irradiated (400Rad) and injected i.v. with male Rag2^-/-^GFP^+^ BM cells. Three days later they received 1.5x10^6^ male-specific Mo TCR-Tg naïve Marilyn CD4^+^ T cells alone, 1.5x10^6^ male specific Mo TCR-Tg CD8^+^ T cells alone or both T cell populations together. HY specific TCR Tg CD4^+^ and CD8^+^ cells were studied as described in Fig 5A and 5B. **Upper panels** show mice injected with female (Ag^-^) Rag2^-/-^ GFP^+^ BM and **lower panels** with male (Ag^+^) Rag2^-/-^ GFP^+^ BM cells and CD4^+^ T cells (left); CD8^+^ T cells (middle); or both T cell populations (right). Scale bars correspond to 50 μm.
(TIF)

S1 Video Motility behaviour of individual T cells. Collapsed 4D images depicting CD8^+^ T cells (red), CD4^+^ T cells (blue) in the spleen red pulp 4–6 hours after T cell transfer. The small blue spots are cell debris observed soon after T cell transfer. Trajectories of motile T cells are shown in white lines, the time as hours:minutes:seconds scale bar, 40 μm.
(MOV)

S2 Video Motility behaviour of individual Ag^+^APCs. Collapsed 4D images depicting Ag^+^APCs (green), CD8^+^ (red), CD4^+^ (blue) T cells in spleen red pulp 4–6 hours after T cell transfer. Trajectories of motile APCs are shown in white lines, time as hours:minutes:seconds, scale bar, 20 μm.
(MOV)

S3 Video Motility behavior of individual T cells in the absence of the HY antigen. Collapsed 4D images depicting CD8^+^ T cells (red), CD4^+^ T cells (blue) in the spleen red pulp 24 hours after T cell transfer in the absence of the HY antigen. Trajectories of motile T cells are shown in white lines, the time as hours:minutes:seconds scale bar, 20 μm.
(MOV)

S4 Video Motility behaviour of a CD8^+^/Ag^+^DC complex. Collapsed 4D images depicting CD8 T cell (red)/Ag^+^DC (green) stable contact in the spleen red pulp 4–6 hours after T cell transfer. The trajectories of these cells are shown in white lines, time as hours:minutes:seconds, scale bar, 15 μm.
(MOV)

S5 Video Dynamics of the formation of ternary complex. Collapsed 4D images depicting 2 CD8^+^ T cells (red) one Ag^+^ DC (green) and a CD4^+^ T cell (blue), in spleen red pulp 4–6 hours after T cell transfer. The trajectories of these cells are shown in white lines, time as hours:minutes:seconds, scale bar, 20 μm. See also Fig 4 legend.
(MOV)

S6 Video Dynamics of the formation of ternary complex. Collapsed 4D images depicting CD8^+^ T cells (red), Ag^+^DC (green) and CD4^+^ T cells (blue), in spleen red pulp 4–6 hours after T cell transfer. Time is indicated as hours:minutes:seconds, scale bar, 20 μm. See also Fig 4 legend.
(MOV)

S1 Note Confocal and TPLSM *in vivo* imaging studies. The main advantage of confocal versus *in vivo* imaging studies is that in the former we can study multiple sections, i.e., the whole spleen; while in the later we are restricted to a single plan, and to a restricted area. For imaging studies we select an area where CD4^+^, CD8^+^ and DCs are present using a low magnification, but this is insufficient to visualize interactions properly. We use a higher magnification that (with luck) has interactions. Once an interaction is detected it must be followed until the end of the imaging period, we cannot change the field of view. Moreover, in contrast to the LNs that are much smaller than the spleen, the whole area to be visualized in the spleen is much larger and cells may be dispersed. Therefore, the number of complexes we can study using TPLSM is necessarily restricted. Confocal imaging can complement our TPLSM imaging studies.
(DOCX)

S2 Note The behavior of T cells and DCs in the spleen. The behavior of T cells and DCs in the spleen must be different from that in the LNs. In contrast to the LN, spleens do not have HEV surrounded by DC and lack migratory DCs that transport the Ag; T cells circulatory behavior is totally different. The location of Ag encounters must also differ. The spleen is specialized in the clearing pathogens that enter the organ via blood and not via lymphatics, as it is the case of the majority of cases of LN immunizations.
(DOCX)
