# Supplementary figures and images for: CD4/CD8/Dendritic cell complexes in the spleen: CD8+ T cells can directly bind CD4+ T cells and modulate their response
Source: PLoS One. 2017 Jul 7;12(7):e0180644. doi: 10.1371/journal.pone.0180644 (PMC5501581; doi:10.1371/journal.pone.0180644)

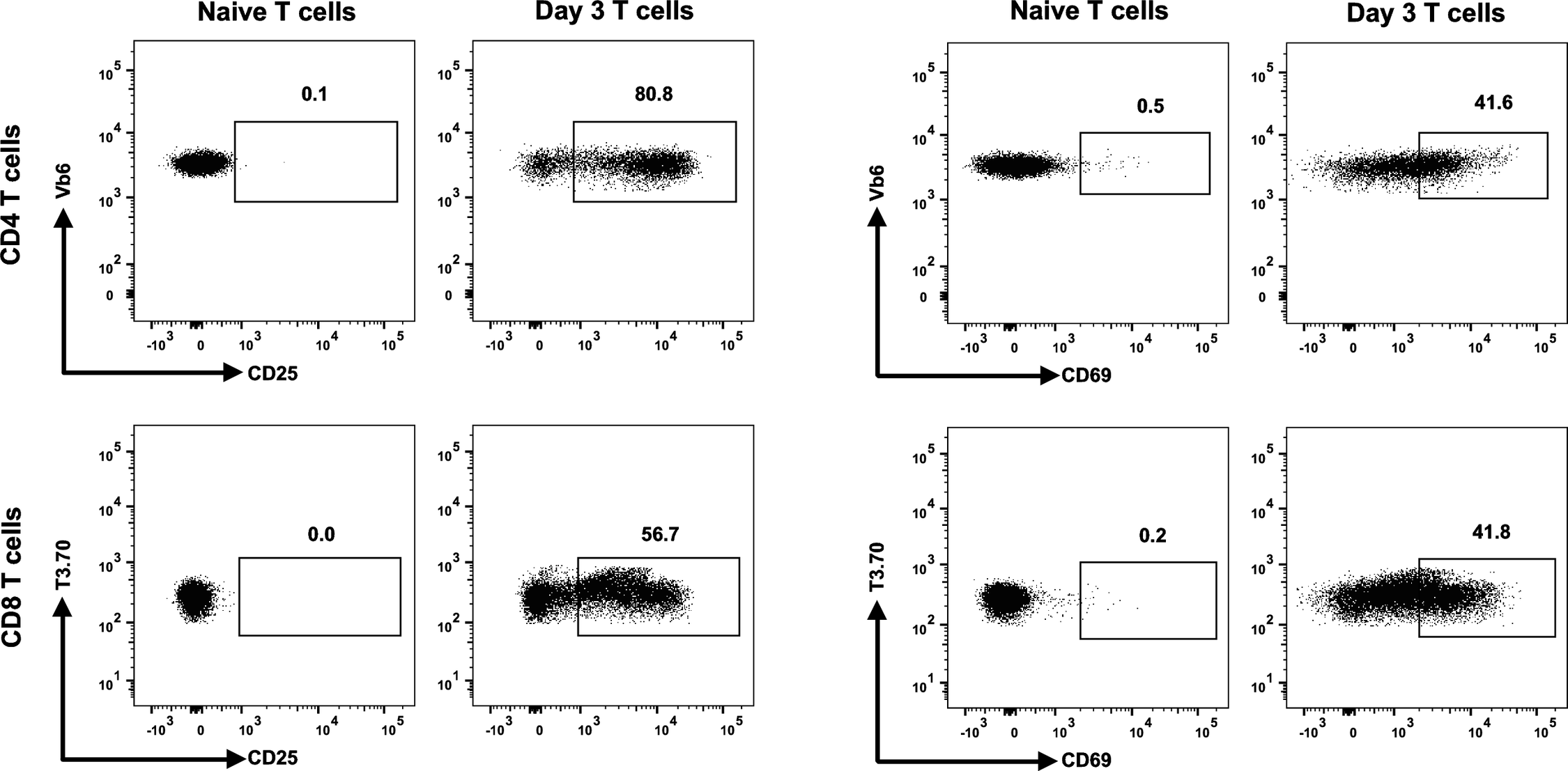

Supplement: S1 Fig — CD4+ Vβ6+ T cells (upper panels) and CD8+ T3.70+ T cells (lower panels) from male-specific TCR Tg mice were studied for CD25 and CD69 expression. Most left dot plots show the expression of CD25 in naïve T cells; left dot plots show the expression of CD25 in T cells on day 3 after transfer into Rag2-/- hosts that were irradiated and reconstituted with male (Ag+) Rag2-/- GFP+ BM cells; right dot plots show CD69 expression in naïve T cells from male-specific Tg mice; most right dot plots show CD69 expression in T cells on day 3 after transfer into Rag2-/- hosts that were irradiated and reconstituted with male (Ag+) Rag2-/- GFP+ BM cells. (TIF) [file pone.0180644.s001.tif]

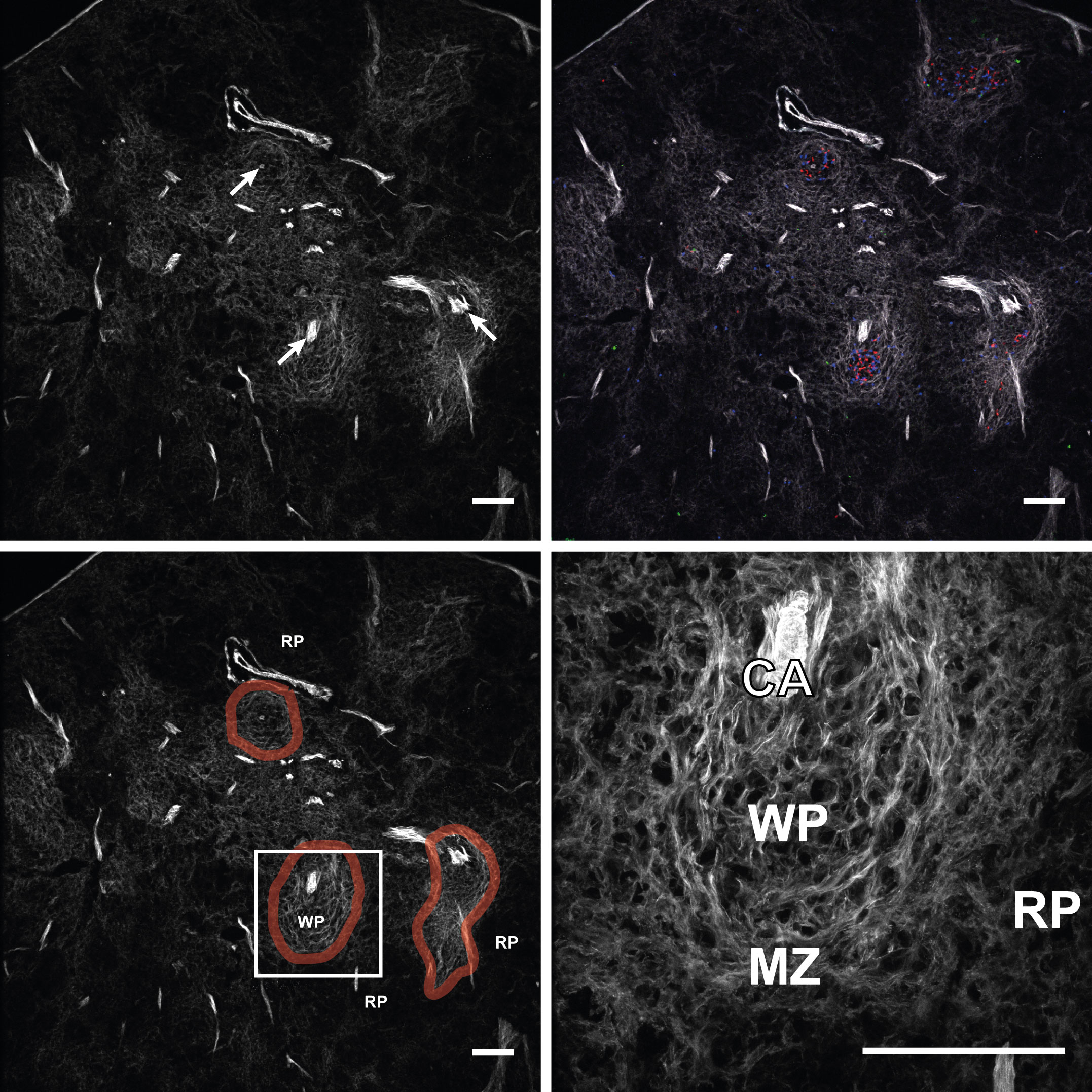

Supplement: S2 Fig — Images show cross-sections of the spleen from Fig 5A. The spleen stroma is labeled with Alexa Fluor 635 phalloidin, CD4+ T cells labeled in blue and CD8+ T cells in red. Upper panels, left: phalloidin staining alone, arrows indicating the central arteriole (CA) of the white pulp (WP); right: the same cross-section showing labeled T cells accumulating in close proximity to the CA, defining the T cell zone or PALS. Scale bars corresponds to 100μm. Lower Panels, left: orange shading indicates the border of the WP and the red pulp (RP), and is defined as the marginal zone (MZ), square denotes the area magnified in right image; right: higher magnification view defining the white pulp (WP), central arteriole (CA), marginal zone (MZ) and red pulp (RP). Scale bars correspond to 100μ. (TIF) [file pone.0180644.s002.tif]

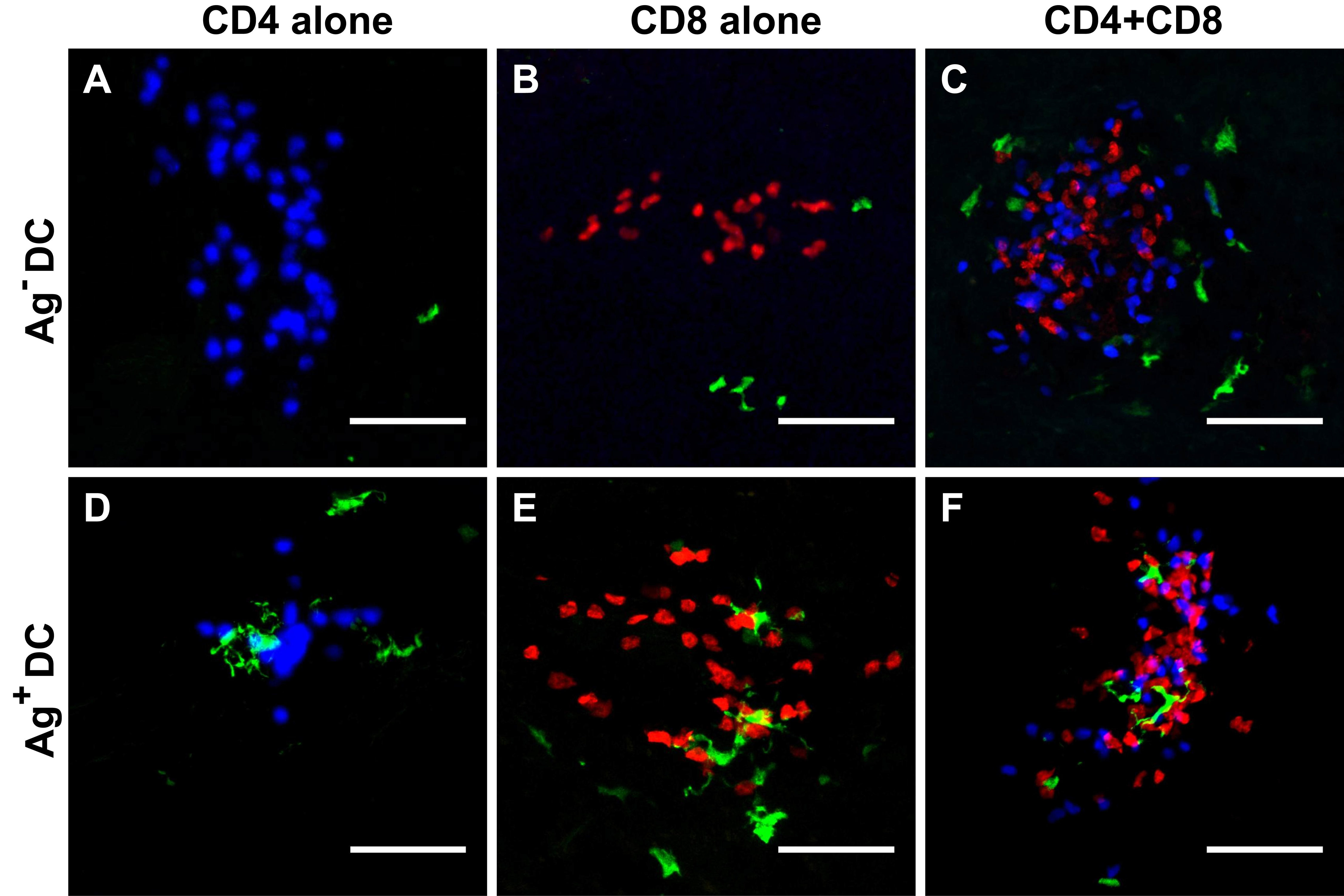

Supplement: S3 Fig — Rag2-/- female mice were irradiated (400Rad) and injected i.v. with male Rag2-/-GFP+ BM cells. Three days later they received 1.5x10^6^ male-specific Mo TCR-Tg naïve Marilyn CD4+ T cells alone, 1.5x10^6^ male specific Mo TCR-Tg CD8+ T cells alone or both T cell populations together. HY specific TCR Tg CD4+ and CD8+ cells were studied as described in Fig 5A and 5B. Upper panels show mice injected with female (Ag-) Rag2-/- GFP+ BM and lower panels with male (Ag+) Rag2-/- GFP+ BM cells and CD4+ T cells (left); CD8+ T cells (middle); or both T cell populations (right). Scale bars correspond to 50 μm. (TIF) [file pone.0180644.s003.tif]
