# Supplementary material for: CD4/CD8/Dendritic cell complexes in the spleen: CD8+ T cells can directly bind CD4+ T cells and modulate their response
Source: PLoS One. 2017 Jul 7;12(7):e0180644. doi: 10.1371/journal.pone.0180644 (PMC5501581; doi:10.1371/journal.pone.0180644)
Supplement: S1 Note — The main advantage of confocal versus in vivo imaging studies is that in the former we can study multiple sections, i.e., the whole spleen; while in the later we are restricted to a single plan, and to a restricted area. For imaging studies we select an area where CD4+, CD8+ and DCs are present using a low magnification, but this is insufficient to visualize interactions properly. We use a higher magnification that (with luck) has interactions. Once an interaction is detected it must be followed until the end of the imaging period, we cannot change the field of view. Moreover, in contrast to the LNs that are much smaller than the spleen, the whole area to be visualized in the spleen is much larger and cells may be dispersed. Therefore, the number of complexes we can study using TPLSM is necessarily restricted. Confocal imaging can complement our TPLSM imaging studies. (DOCX) [file pone.0180644.s010.docx]

**S1 Note. Confocal and TPLSM *in vivo* imaging studies.** The main advantage of confocal versus *in vivo* imaging studies is that in the former we can study multiple sections, i.e., the whole spleen; while in the later we are restricted to a single plan, and to a restricted area. For imaging studies we select an area where CD4^+^, CD8^+^ and DCs are present using a low magnification, but this is insufficient to visualize interactions properly. We use a higher magnification that (with luck) has interactions. Once an interaction is detected it must be followed until the end of the imaging period, we cannot change the field of view. Moreover, in contrast to the LNs that are much smaller than the spleen, the whole area to be visualized in the spleen is much larger and cells may be dispersed. Therefore, the number of complexes we can study using TPLSM is necessarily restricted. Confocal imaging can complement our TPLSM imaging studies.
