# Supplementary material for: CD4/CD8/Dendritic cell complexes in the spleen: CD8+ T cells can directly bind CD4+ T cells and modulate their response
Source: PLoS One. 2017 Jul 7;12(7):e0180644. doi: 10.1371/journal.pone.0180644 (PMC5501581; doi:10.1371/journal.pone.0180644)
Supplement: S2 Note — The behavior of T cells and DCs in the spleen must be different from that in the LNs. In contrast to the LN, spleens do not have HEV surrounded by DC and lack migratory DCs that transport the Ag; T cells circulatory behavior is totally different. The location of Ag encounters must also differ. The spleen is specialized in the clearing pathogens that enter the organ via blood and not via lymphatics, as it is the case of the majority of cases of LN immunizations. (DOCX) [file pone.0180644.s011.docx]

**S2 Note**. **The behavior of T cells and DCs in the spleen.** The behavior of T cells and DCs in the spleen must be different from that in the LNs. In contrast to the LN, spleens do not have HEV surrounded by DC and lack migratory DCs that transport the Ag; T cells circulatory behavior is totally different. The location of Ag encounters must also differ. The spleen is specialized in the clearing pathogens that enter the organ via blood and not via lymphatics, as it is the case of the majority of cases of LN immunizations.
